# Lipids in the Physiopathology of Hereditary Spastic Paraplegias

**DOI:** 10.3389/fnins.2020.00074

**Published:** 2020-02-28

**Authors:** Frédéric Darios, Fanny Mochel, Giovanni Stevanin

**Affiliations:** ^1^Sorbonne Université, Paris, France; ^2^Inserm, U1127, Paris, France; ^3^CNRS, UMR 7225, Paris, France; ^4^Institut du Cerveau et de la Moelle Epinière, Paris, France; ^5^National Reference Center for Neurometabolic Diseases, Pitié-Salpêtrière University Hospital, Assistance Publique-Hôpitaux de Paris, Paris, France; ^6^Equipe de Neurogénétique, Ecole Pratique des Hautes Etudes, PSL Research University, Paris, France

**Keywords:** phospholipids, sphingolipids, fatty acids, cholesterol, metabolism, myelin, lysosome

## Abstract

Hereditary spastic paraplegias (HSP) are a group of neurodegenerative diseases sharing spasticity in lower limbs as common symptom. There is a large clinical variability in the presentation of patients, partly underlined by the large genetic heterogeneity, with more than 60 genes responsible for HSP. Despite this large heterogeneity, the proteins with known function are supposed to be involved in a limited number of cellular compartments such as shaping of the endoplasmic reticulum or endolysosomal function. Yet, it is difficult to understand why alteration of such different cellular compartments can lead to degeneration of the axons of cortical motor neurons. A common feature that has emerged over the last decade is the alteration of lipid metabolism in this group of pathologies. This was first revealed by the identification of mutations in genes encoding proteins that have or are supposed to have enzymatic activities on lipid substrates. However, it also appears that mutations in genes affecting endoplasmic reticulum, mitochondria, or endolysosome function can lead to changes in lipid distribution or metabolism. The aim of this review is to discuss the role of lipid metabolism alterations in the physiopathology of HSP, to evaluate how such alterations contribute to neurodegenerative phenotypes, and to understand how this knowledge can help develop therapeutic strategy for HSP.

## Introduction

Hereditary spastic paraplegias (HSP) are a group of rare neurodegenerative diseases characterized by weakness of lower limbs and spasticity (Harding, 1983). These symptoms are due to the degeneration of the long axons of the neurons from the motor cortex. Since the longest axons seemed to be more sensitive to neurodegeneration, it was proposed that altered intracellular trafficking could underlie the physiopathology of HSP (Soderblom and Blackstone, 2006). HSP are clinically heterogeneous, and complex forms of HSP are associated with various other neurological symptoms such as cognitive impairment or ataxia, due to degeneration of other neuronal populations. This clinical heterogeneity is partly underlined by the genetic heterogeneity of this group of diseases. Indeed, in the last decade, the emergence of new genetic tools allowed the identification of many genes responsible for HSP (Boutry et al., 2019a). Among the recently identified genes, many encode enzymes that are directly implicated in the metabolism of lipids. Since brain is mainly composed of lipids, identification of such lipid-related pathways opens new perspectives to evaluate the physiopathology of HSP. Importantly, beside the identification of genes encoding lipid-modifying enzymes, mutations in genes responsible for HSP and coding proteins involved in membrane trafficking can also alter lipid homeostasis in some subcellular compartments. The formation of myelin, the most prominent lipidic structure in the brain, also appears to play a critical role in the maintenance of axon in several forms of HSP. Therefore, it becomes evident that lipids are of critical importance for the physiopathology of almost all HSP. In this review, we propose in a first part an overview of the various metabolic pathways that are altered by mutations in genes responsible for HSP and HSP-related disorders such as leukodystrophies presenting with spasticity. In a second part, we examine the consequences of altered lipid metabolism on cellular functions to highlight various physiopathological pathways that could be altered in the different forms of HSP.

## Alterations of Lipid Metabolism Due to HSP-Causing Mutations

The emergence of next-generation sequencing in the last decade allowed the identification of many genes responsible for HSP. Among all the newly identified genes, a quarter of them are encoding enzymes that are directly involved in the metabolism of lipids (Table 1). It appears that all the main classes of lipids could be implicated in the physiopathology of HSP.

**TABLE 1:** Genes responsible for HSP encoding enzymes of the metabolism of lipids.

**Gene**	**Protein name**	**Function**
**Cholesterol**		
*CYP7B1* (*SPG5*)	Cytochrome P450-7B1	Degradation of oxysterols
*CYP27A1* (CTX)	Cytochrome p450-7A1	Degradation of oxysterols
**Phospholipids**		
*EPT1/SelenoI*	Ethanolaminephospho-transferase 1	Phosphatidylethanolamine synthesis, plasmalogens synthesis
*SERAC1*	SERAC1	Phosphatidylglycerol remodeling
*PNPLA6/NTE* (SPG39)	Patatin-like phospholipase domain containing 6/Neuropathy target esterase	Phospholipase A2
*PLA2G6*	Group VI phospholipase A2, iPLA2b	Phospholipase A2, release of docosahexaenoic acid from phospholipids
*DDHD1* (SPG28)	DDHD1	Phospholipase A1, hydrolysis of PI, PA, PS
*DDHD2* (SPG54)	DDHD2	Phospholipase A1, hydrolysis of PA; TAG lipase
*CYP2U1* (SPG49/56)	CYP2U1	Cytochrome P450, hydroxylation of fatty acids, oxidation of N-arachidonoylserotonin
**Glycosphingolipids**		
*B4GALNT1* (SPG26)	β-1,4-N-acetyl-galactosaminyltransferase	Synthesis of complex gangliosides
*GBA2* (SPG46)	β-Glucosidase 2	Non-lysosomal glucosylceramidase
*FA2H* (SPG35)	Fatty acid-2 hydroxylase	Formation of 2-hydroxy fatty acids incorporated into galactosylceramide
*SLC33A1* (SPG42)	Acetyl-coA transporter 1	O-acetylation of complex gangliosides GD3 and GT3?
*GALC* (Krabbe)	Galactosylcerebrosidase	Degradation of galactosylceramide
*ARSA* (MLD)	Arylsulfatase A	Degradation of sulfatides
**Fatty acids**		
*ABCD1* (X-ALD)	Adrenoleukodystrophy protein (ADLP)	VLCFA import into peroxisomes
*ELOVL1*	Fatty acid Elongase 1	Elongation of fatty acids
*ELOVL4*	Fatty acid Elongase 4	Elongation of fatty acids
*ALDH3A2* (SLS)	Aldehyde Dehydrogenase 3 Family Member A2	Aldehyde Dehydrogenase

*PI: phosphatidylinositol, PA: phosphatidic acid; PS: phosphatidylserine; TAG: triacylglycerols.*

### Cholesterol

Cholesterol is a lipid highly enriched in the brain, as this organ contains about 20% of total body cholesterol (Dietschy and Turley, 2004). Most of the brain cholesterol enters in the composition of myelin, but cholesterol is also found in the membranes of glial and neuronal cells where it is actively trafficked.

#### Cholesterol Hydroxylation

The identification of mutations in cytochrome P450-7B1 (*CYP7B1*) in SPG5 patients (Tsaousidou et al., 2008) was the first indication that lipid metabolism could play a role in the physiopathology of HSP. SPG5 patients often present with pure HSP, but some of them may present with complicated forms of HSP and mild white matter abnormalities (Goizet et al., 2009; Marelli et al., 2018). *CYP7B1* encodes a cytochrome P450 7α-hydroxylase responsible for the degradation of oxysterols. Consequently, loss of CYP7B1 leads to the accumulation of oxysterols such as 25-hydroxycholesterol, 26-hydroxycholesterol, 27-hydroxysterol, and 3β-hydroxy-5-cholestenoic acid (3β-CA) in serum and cerebrospinal fluid of SPG5 patients (Schüle et al., 2010; Theofilopoulos et al., 2014; Marelli et al., 2018). 3β-CA was demonstrated to exert toxic effects on rodent oculomotor neurons *in vitro* and zebrafish motor neurons *in vivo via* activation of liver X receptors (LXRs) (Theofilopoulos et al., 2014). In addition, an *in vitro* study using NSC-34 cell line and neurons derived from human induced pluripotent stem (iPS) cells showed that oxysterols and 3β-CA had a cytotoxic activity. In that study, 25-OHC and 27-OHC were harmful at concentrations comparable to levels measured in serum of SPG5 patients (Schöls et al., 2017). However, in both studies, the toxicity observed required levels of oxysterols that were much higher than the levels detected in the cerebrospinal fluid of SPG5 patients. The cytotoxic action of oxysterols are mainly due to their incorporation into the natural lipid bilayer, where they can change the interaction between molecules (Mitomo et al., 2009) and thus alter membrane properties. In both studies investigating the toxic accumulation of CYP7B1 substrates, oxysterols added to the medium likely partitioned between medium and membranes, and their real concentration in membranes were not analyzed. Furthermore, the concentration of oxysterols that need to be reached in the membranes to induce a deleterious effect are not known, and further investigations are required to evaluate the mechanisms underlying neurodegeneration in SPG5 patients. Based on the hypothesis that oxysterols are neurotoxic, two concomitant therapeutic trials were conducted in SPG5 patients with decreased plasma oxysterols as the primary outcome measure (Schöls et al., 2017; Marelli et al., 2018). In both short-term phase II trials, atorvastatin significantly decreased plasma 27-OHC (Schöls et al., 2017; Marelli et al., 2018). Furthermore, treatment with chenodeoxycholic acid (CDCA) restored bile acids profile in SPG5 patients (Marelli et al., 2018). However, the clinical benefit of such metabolic intervention remains to be established and surrogate markers are needed due to the very slowly progressive course and the rarity of this disease. Another clinical trial levels using monoclonal antibodies that inhibit proprotein convertase subtilisin-kexin type 9 (PCSK9) to reduce cholesterol levels has recently been initiated (Chen, 2019). It will monitor levels of 27-OHC as a primary outcome.

Another HSP related to defective cholesterol hydroxylation is cerebrotendinous xanthomatosis (CTX) due to *CYP27A1* mutations, which encodes the mitochondrial cytochrome P450 enzyme sterol 27-hydroxylase. Deficiency in this enzyme interferes with sterol intermediates in the alternative bile acid pathway. More specifically, CTX is associated with reduced synthesis of 27-OHC and CDCA, as well as the shunting of sterol intermediates into the microsomal pathway for cholic acid formation (Salen et al., 1991). CTX is also characterized by high production of cholestanol, which accumulates in various tissues, as well as increased levels of bile alcohols in urine (Berginer et al., 1984). Evidence that cholestanol may be neurotoxic is supported by the finding of cholestanol deposition and apoptosis in neuronal cells in the cerebellum of rats fed a 1% cholestanol diet (Inoue et al., 1999). As the influx of 27-OHC may be involved in brain cholesterol homeostasis, the lack of 27-OHC may also impact cholesterol synthesis in the brain (Mignarri et al., 2016). About 60% of CTX patients (Wong et al., 2018) present with a complex form of HSP that includes systemic (infantile cholestasis, juvenile-onset cataracts, Achilles tendon xanthomas, chronic diarrhea, and osteoporosis) and/or neuropsychiatric symptoms (learning disability and/or autism spectrum disorder, cerebellar ataxia, peripheral neuropathy, parkinsonism, dementia, and psychiatric disturbances) (Nie et al., 2014). Importantly, there is a critical therapeutic window in most CTX patients before the onset of disabling neuropsychiatric symptoms (Yahalom et al., 2013). CDCA remains the treatment of choice in CTX as it down-regulates CYP27A1, restores the imbalance between CDCA and cholic acid, and is the only drug that has shown beneficial effects on neurological symptoms so far (Nie et al., 2014; Salen and Steiner, 2017). Several studies have emphasized that the response to treatment strongly depends on when CDCA is initiated (Amador et al., 2018; Duell et al., 2018; Stelten et al., 2019). In a cohort of 56 Dutch CTX patients treated by CDCA with a median follow-up time of 8 years, neurological symptoms, assessed by the modified Rankin Scale and Expanded Disability Status Scale (EDSS) scores, disappeared in all patients who were diagnosed before the age of 24 and treated since (Stelten et al., 2019). Furthermore, treatment prevented the development of new neurological symptoms during the follow-up period (Stelten et al., 2019).

#### Alteration of Cholesterol Synthesis or Trafficking in HSP

Oxysterols are present at very low levels compared to cholesterol (Wnętrzak et al., 2017), and alterations of cholesterol synthesis or trafficking are induced by the loss of function of several genes responsible for HSP.

For example, loss of function of Erlin1 and Erlin2, mutated in SPG62 and SPG18, respectively, are responsible for early onset autosomal recessive HSP associated with cognitive impairment (Alazami et al., 2011; Novarino et al., 2014). Autosomal dominant mutations in *Erlin2*, associated with SPG37, are also implicated in less severe forms of motor neuron diseases (Rydning et al., 2018; Stevanin et al., 2019). Erlin1 and Erlin2 form a heteromultimeric complex of ∼2 MDa (Pearce et al., 2009). This complex is localized in the lumen of the ER and is anchored to cholesterol-rich lipid domains by the N-terminus of the proteins (Browman, 2006). Downregulation of Erlin1 and 2 was shown to activate the sterol regulatory element binding protein (SREBP) transcription factors, leading to upregulation of their target genes. This increased cholesterol content in cells and cells containing cytoplasmic lipid inclusions stained with Oil Red O (Huber et al., 2013). However, the relevance of this function for the physiopathology of HSP has not been evaluated yet.

Loss of spatacsin, due to mutations in *SPG11*, is responsible for a complicated form of HSP where spasticity is often associated with cognitive impairment and lower motor neuron degeneration. White matter anomalies may be detected on brain MRI (Hehr et al., 2007; Stevanin et al., 2007, 2008). Spatacsin is implicated in the autophagic lysosome reformation, a mechanism allowing the formation of tubules in autolysosomes at the end of the autophagy process in order to recycle lysosome membrane (Chang et al., 2014). Recently, it was shown that the formation of tubules on lysosomes mediated by spatacsin allows the recycling of cholesterol from lysosomes. Loss of spatacsin led to an accumulation of cholesterol in lysosomes and decreased cholesterol content in plasma membrane (Boutry et al., 2019b), which could affect membrane properties and function. This highlights that impaired cholesterol trafficking and not only cholesterol overload in lysosomes could contribute to the pathology. However, the contribution of this mechanism to neuronal death is still not known.

### Alteration of Phospholipid Synthesis and Degradation in Some HSP Entities

**FIGURE 1:**
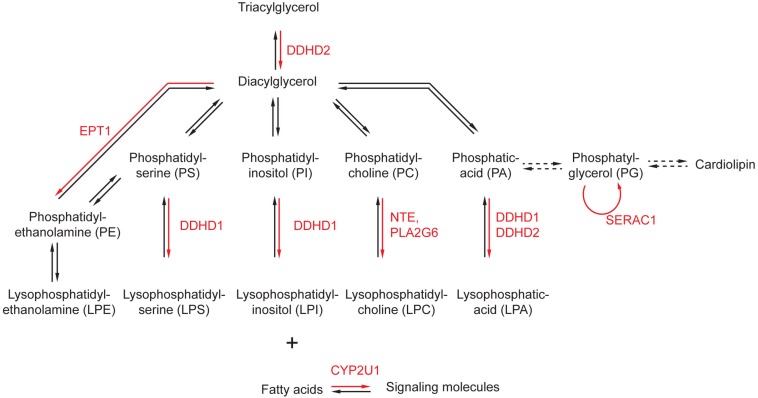
Alteration of phospholipid metabolism by genes implicated in the physiopathology of HSP. The main metabolic pathways affecting phospholipids and phospholipid-related molecules are represented. The enzymes affected by mutations in genes responsible for HSP are highlighted in red. Dashed arrows represent conversion requiring more than one enzymatic reaction.

Phospholipids are the main components of cellular membranes. They are composed of a glycerol backbone to which two fatty acids are attached as esters. The third alcohol group of the glycerol is attached to a phosphoric acid that can associate various hydrophilic moieties, determining the nature of the phospholipid (phosphatidyl-choline, phosphatidyl inositol, etc.) (Figure 1). Several enzymes implicated in the metabolism of phospholipids have been associated with HSP or related disorders.

#### Altered Synthesis of Phospholipids

Mutations in *EPT1/SELENOI* have been found in patients presenting an early onset complicated HSP with white matter abnormalities detected on brain MRI (Ahmed et al., 2017; Horibata et al., 2018). EPT1 is an ethanolamine transferase implicated in the synthesis of phosphatidylethanolamine (PE), the second most abundant phospholipid in membranes. Analysis of a patient’s fibroblasts as well as *EPT1* knockout Hela cells showed decreased PE biosynthesis, as well as an imbalance in plasmalogens (increased plasmanyl-phosphatidylcholine and decreased plasmenyl-phosphatidylethanolamine) (Horibata et al., 2018). Of note, plasmenyl-phosphatidylethanolamine represents more than 50% of ethanolamine glycerophospholipids in the brain (Braverman and Moser, 2012), which highlights why the loss of EPT1 activity may lead to such severe brain dysfunction.

Mutations in *SERAC1* were known to cause the autosomal recessive MEGDEL syndrome, characterized by infantile onset symptoms including feeding problem, liver failure, deafness, dystonia, encephalopathy, spasticity, and Leigh-like syndrome (Wortmann et al., 2012). Recently, mutations in *SERAC1* were found in a family presenting juvenile onset complicated HSP (Roeben et al., 2018). SERAC1 is a phospholipid remodeling enzyme that converts phosphatidylglycerol 34:1 (PG34:1) into phosphatidylglycerol 36:1 (PG36:1). Families presenting with an HSP phenotype had a milder change in the PG34:1/PG36:1 ratio compared to patients with MEGDEL syndrome (Roeben et al., 2018), suggesting phenotype–genotype correlation.

Mutations in *PGAP1* have been identified in one family of complex HSP (SPG67) (Novarino et al., 2014), but also in cases of intellectual disability and encephalopathy (Murakami et al., 2014). *PGAP1* encodes an ER protein that promotes the deacylation of inositol in the glycosylphosphatidylinositol (GPI)-anchored proteins (Tanaka et al., 2004). During their synthesis, GPI-anchored proteins are localized in the lumen of the ER. The remodeling of GPI anchor by PGAP1 is required for the sorting of GPI-anchored proteins to ER exit sites, allowing their export out of the ER (Fujita et al., 2011).

#### Degradation of Glycerophospholipids

Several enzymes with phospholipase activity have been associated with HSP. Mutations in the genes encoding two calcium-independent phospholipases, NTE/PNPLA6 and PLA2G6, are responsible for a variety of neurodegenerative disorders. Mutations in neuropathy target esterase (*NTE/PNPLA6*) are responsible for autosomal recessive HSP (SPG39) (Rainier et al., 2008), but also other neurological syndromes combining ataxia, spasticity, retinal degeneration, and/or hypogonadism (Synofzik et al., 2014). PLA2G6 associated neurodegeneration is a group of heterogeneous neurodegenerative disorders, including infantile neuroaxonal dystrophy, neurodegeneration with brain iron accumulation, parkinsonism, and HSP. Both enzymes hydrolyze the sn-2 acyl chain of phospholipids. NTE/PNPLA6 seems to have a privileged affinity for phosphatidylcholine, as its levels were increased in the brain of *nestin-cre:NTEfl/fl* mice (Read et al., 2009). The product of *PLA2G6* mediates the release of docosahexaenoic acid from brain phospholipids (Green et al., 2008), suggesting that different actions of phospholipases can contribute to the physiopathology of HSP.

Another group of phospholipases associated with the physiopathology of HSP are DDHD containing enzymes DDHD1 and DDHD2 (Schuurs-Hoeijmakers et al., 2012; Tesson et al., 2012). Mutations in *DDHD1* are responsible for pure or complex HSP (SPG28), sometimes associated with brain iron accumulation (Dard et al., 2017). *DDHD2* is mutated in SGP54, which is usually characterized by very early onset spastic paraplegia, intellectual disability, and white matter abnormalities (Schuurs-Hoeijmakers et al., 2012), but adult onset forms have been reported for both DDHD1 and DDHD2 (Doi et al., 2015; Dard et al., 2017). As a unique finding in DDHD2, proton magnetic resonance spectroscopy revealed an abnormal lipid peak in the basal ganglia and thalamus area (Schuurs-Hoeijmakers et al., 2012). *In vitro* experiments proposed that DDHD1 and DDHD2 have a phosphatidic acid-preferring phospholipase A1 action, hydrolyzing the sn-1 acyl chain (Higgs et al., 1998; Inoue et al., 2012). These results were challenged by lipidomic analysis of knockout mouse models for these two genes. Knockout of *Ddhd2* led to accumulation of triacylglycerol, and recombinant DDHD2 was revealed to have a triacylglycerol-lipase activity (Inloes et al., 2014). Recently, lipidomic analysis of the brain of *Ddhd1* knockout mice showed that phosphatidylinositol and phosphatidylserine are the main physiological substrates of DDHD1 (Inloes et al., 2018). It is conceivable that DDHD1 and DDHD2 have different substrates depending on the cell type that is investigated.

#### Metabolism of Phospholipid and Cellular Signaling

The metabolism of phospholipids can contribute to signaling pathways by modifying various lipid species. One of the most studied lipid signaling pathway relies on the phosphorylation state of phosphoinositides (Wang et al., 2019). Interestingly, loss of Ddhd1 in mice led to striking remodeling of phosphoinositides in the brain (Inloes et al., 2018). Furthermore, spastizin (*ZFYVE26*) mutated in SPG15 (Hanein et al., 2008) has a FYVE domain that mediates interaction with phosphatidylinositol-3-phosphate (PI3P). The ability of the spastizin FYVE domain to bind PI3P is required for addressing protein to lysosomes (Chang et al., 2014), even if the content of PI3P in this subcellular compartment is poorly characterized. Deletion of the FYVE domain or point mutation abolishing the interaction with PI3P impaired the localization of spastizin to lysosomes (Chang et al., 2014). Loss of phosphatidylinositol 4-kinase 2alpha activity in mouse caused neurological symptoms resembling HSP (Simons et al., 2009). In this mouse model, the authors revealed neurodegeneration that was associated to accumulation of lipofuscin-like material, similar to the observation made in *Spg15* knockout mice (Khundadze et al., 2013). Although mutations in phosphatidylinositol 4-kinase 2 alpha have not been identified in HSP patients so far, this illustrates the role of signaling phosphoinositides in the physiopathology of HSP.

It is important to note that the phospholipases A1 or A2 may release lysosphospholipids such as lysophosphatidic acid or lysophosphatidyl-inositol and some of them play an important a role in cellular signaling (Yung et al., 2015). The phospholipases A2 hydrolyze phospholipids in the sn-2 position, releasing a fatty acid that is often unsaturated. Such fatty acid can be hydroxylated by the cytochrome P450 *CYP2U1* that is mutated in the early onset form of complex HSP SPG49 (HUGO) or SPG56 (OMIM) (Chuang et al., 2004; Tesson et al., 2012). Oxidation of unsaturated fatty acids can produce a variety of signaling molecules, but their identities and roles are still to be uncovered. The role of such signaling molecules in the physiopathology of HSP has not been investigated so far, and it may open new avenues to identify targets of therapeutic interest for these diseases.

### Alteration of Sphingolipid Metabolism in HSP

**FIGURE 2:**
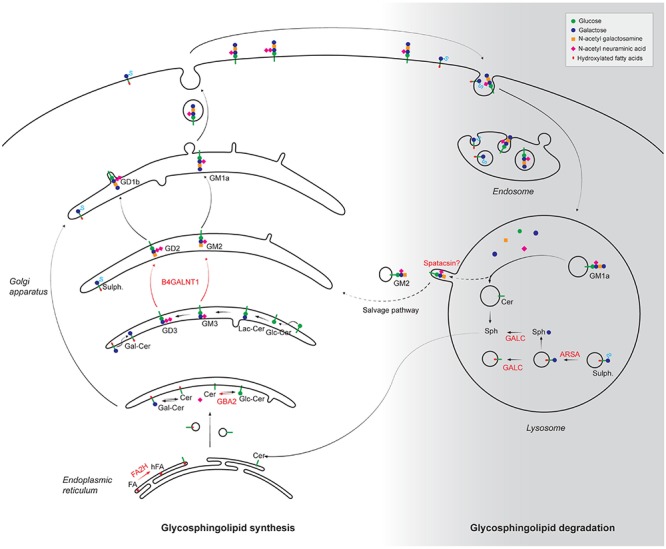
Alteration of synthesis and degradation pathways of glycosphingolipids in the physiopathology of HSP. The different steps of glycosphingolipid biosynthesis occur in the endoplasmic reticulum and the Golgi apparatus by progressive addition of the molecules indicated in the top right panel. Following internalization by endocytosis, plasma membrane glycosphinglipids are progressively degraded in lysosome into basic building blocks. The products of HSP-mutated genes that are implicated in the metabolism of glycosphingolipids are highlighted in red. FA, fatty acids; hFA, hydroxylated fatty acids; Cer, Ceramide; Gal-Cer, Galatosyl-ceramide; Glc-Cer, Glucosyl-ceramide; Lac-Cer, Lactosyl-ceramide; Sulf: Sulfatides; Sph, Sphingosine; FA2H, fatty acid-2-hydroxylase; GBA2, β-glucosidase 2; B4GALNT1, β-1,4-N-acetyl-galactosaminyltransferase; GALC, Galactosyl-cerebrosidase; ARSA, Arylsulfatase A. It has been proposed that some gangliosides could be recycled from the lysosomes using a salvage pathway. Whether spatacsin could be implicated in such pathway is still unclear.

Sphingolipids are a group of lipids derived from the sphingosine backbone. They are synthesized in the ER and the Golgi apparatus, and are degraded by lysosomes (Figure 2). Alteration of both biosynthetic and degradative pathways of sphingolipids have been associated with HSP phenotype.

#### Sphingolipid Biosynthesis

Loss-of-function mutations in fatty acid-2-hydroxylase (FA2H) are responsible for the complex form of autosomal recessive HSP (SPG35) (Edvardson et al., 2008) and allelic neurological disorders. A recent study showed that patients with nonsense or missense mutations presented a rather homogeneous phenotype, with a spastic tetraparesis frequently associated with cognitive deficits. Symptoms onset is in early childhood and all patients presented white matter abnormalities on brain MRI (Rattay et al., 2019). FA2H is the enzyme responsible for the formation of 2-hydroxy-fatty acids (Alderson et al., 2004) that are frequently incorporated as N-acyl chain in galactosylceramide (Bowen and Radin, 1968). In mouse models, the loss of function of Fa2h was associated with the absence of sphingolipids containing 2-hydroxylated fatty acids and increased non-hydroxylated galactosylceramide (Zoller et al., 2008).

Gangliosides are synthesized in the ER. These glycosphingolipids are highly enriched at the plasma membranes of neurons and glial cells, notably complex gangliosides such as GM1 and GD1. These molecules serve as coreceptors for many factors. *B4GALNT1* is mutated in SPG26 that is characterized by complex HSP frequently associated with intellectual disability (Boukhris et al., 2013). *B4GALNT1* encodes β-1,4-N-acetyl-galactosaminyl transferase 1 or GM2/GD2 synthase that converts GM3 into GM2 and GD3 into GD2. Accordingly, analysis in fibroblasts of SPG26 patients showed a lack of GM2 associated with increased levels of its precursor GM3 (Harlalka et al., 2013). Knockout of the GM2 synthase in mouse leads to a progressive demyelination and axonal loss that lead to motor dysfunction (Sheikh et al., 1999; Chiavegatto et al., 2000), similar to the symptoms observed in patients.

Other forms of HSP are putatively linked to sphingolipid metabolism, although the roles of the mutated gene products are still elusive. Deficiency in carnitine palmitoyltransferase 1C (CPT1C) accounts for SPG73 and has been associated with sphingolipid metabolism (Rinaldi et al., 2015). This enzyme belongs to a family mediating the trans-esterification of palmitoyl-coA and carnitine, to allow the formation of a molecule that can be transported through membranes, in contrast to palmitoyl-CoA. While CPT1A and CPT1B are enriched in mitochondria and allow the transport of acyl-CoA into mitochondria to undergo β-oxidation, CPT1C is localized in the ER and its carnitine palmitoyl transferase activity is low compared to CPT1A and CPT1B (Casals et al., 2016). In contrast, a lipidomic analysis in the brain of fasted *Cpt1c* knockout mice showed lower levels of ceramide and sphingosine (Carrasco et al., 2012). The molecular mechanisms leading to this change in sphingolipid metabolism are however unknown.

A missense mutation in *SLC33A1* (p. S113R) has been observed in a large family of patients presenting with an autosomal dominant spastic paraplegia (SPG42) (Lin et al., 2008). *SLC33A1* encodes the acetyl-CoA transporter 1 (AT-1) localized in the ER, which has been proposed to play a key role in the acetylation of proteins but also of gangliosides such as O-Ac-GD3 or O-Ac-GT3 (Kanamori et al., 1997). The p. S113R mutant is supposed to disrupt the second transmembrane domain of the protein, thus blocking its function (Lin et al., 2008). A knock-in mouse model expressing the p.S113R mutant at the heterozygous state presents impaired locomotion at the age of 12 months (Liu et al., 2017). This phenotype is associated with a demyelination and alteration of ER and mitochondria in the cell body of neurons. However, whether the synthesis of O-Ac-GD3 or O-Ac-GT3 was decreased in this mouse model has not been investigated. Acetyl-coA has also been associated with fatty acid metabolism (Yoshii et al., 2015), but it has not been investigated whether fatty acid metabolism is impaired in SPG42 patients or in the Spg42 mouse model.

#### Sphingolipid Degradation

Krabbe disease and metachromatic leukodystrophy are two autosomal recessive diseases due to impaired lysosomal degradation of sphingolipids. Both are progressive diseases, usually characterized by spasticity, often related to brain white matter lesions detected on brain MRI—peripheral neuropathy and cognitive impairment (Orsini et al., 2000; Gomez-Ospina, 2006). Krabbe disease is caused by loss of function of galactosylcerebrosidase (GALC) (Rafi et al., 1995). GALC degrades galactosylceramide, the major sphingolipid of myelin, as well as other sphingolipids containing galactose such as psychosine (D-galactosylsphingosine). In the absence of a functional GALC, galactosylceramide can be degraded by GM1 ganglioside β-galactosidase (Kobayashi et al., 1985) and thus do not accumulate in cells of Krabbe disease patients. In contrast, psychosine cannot be metabolized by GM1 ganglioside β-galactosidase and thus accumulates in the brain of Krabbe patients (Svennerholm et al., 1980). In the Twitcher mouse, a natural model of Krabbe disease, a biochemical analysis revealed decreased levels of galactosylceramide and sulfatides at early stages, as well as strong accumulation of psychosine (Igisu et al., 1983; Igisu and Suzuki, 1984). Metachromatic leukodystrophy is due to a deficiency in arylsulfatase A (Gomez-Ospina, 2006). This enzyme mediates the degradation of a class of glycosphingolipids, sulfatides (Mehl and Jatzkewitz, 1968). Consequently, metachromatic leukodystrophy patients present high levels of sulfatides in many tissues, including the brain, and they excrete high levels of sulfatides in urines (Gomez-Ospina, 2006). A mouse model deficient in arylsulfatase presents with early onset accumulation of sulfatides in lysosomes of neurons and oligodendrocytes (Wittke et al., 2004). In both Krabbe disease and metachromatic leukodystrophy, allogenic hematopoietic stem cell transplantation (HSCT) can stabilize/slow-down cerebral demyelinating lesions for pre- or early-symptomatic juvenile or adult patients (van Rappard et al., 2016; Laule et al., 2018).

Krabbe disease and metachromatic leukodystrophy belong to the group of lysosomal storage disorders. However, accumulation of some glycosphingolipids in lysosomes has also been observed in SPG11 models of HSP (Boutry et al., 2018) that is not classically considered as a lysosomal storage disorder. Accumulation of gangliosides in SPG11 was likely not due to impaired function of ganglioside-degrading enzymes, suggesting that the accumulation of gangliosides has another cause. SPG11 is due to the loss of function of spatacsin, a protein that has been implicated in the autophagic lysosome reformation, a mechanism allowing formation of tubule in autolysosomes at the end of the autophagy process to recycle lysosome membrane (Chang et al., 2014). It has been proposed that inhibition of the tubulation in absence of spatacsin could lead to accumulation of gangliosides in lysosomes, which could contribute to the motor dysfunction observed in the mouse model (Branchu et al., 2017; Boutry et al., 2018). Some SPG11 patients are diagnosed as cases of amyotrophic lateral sclerosis or Charcot-Marie tooth disease (Orlacchio et al., 2010; Montecchiani et al., 2016). Of note, the levels of several glycosphingolipids, including gangliosides, were upregulated in the spinal cord of ALS patients, suggesting that they could participate to neurodegeneration in this disease as well (Dodge et al., 2015). SPG11 patients also frequently present parkinsonian features (Anheim et al., 2009). It is interesting to note that the main risk factor identified so far for Parkinson’s disease is the heterozygous loss of *GBA1* (Aharon-Peretz et al., 2004). This gene encodes lysosomal glucosylceramidase, which is a lysosomal enzyme implicated in the degradation of glycosphingolipids downstream the degradation of gangliosides.

Mutations in *GBA1* have not been found in HSP patients, but loss of its paralog *GBA2* is responsible for spastic ataxia with cognitive impairment (SPG46) (Hammer et al., 2013; Martin et al., 2013). GBA2 is a non-lysosomal glucosylceramidase, hydrolyzing glucosylceramide into glucose and ceramide. Most mutations impair the enzymatic function of GBA2 and can thus be considered from an enzymatic perspective as null mutants (Sultana et al., 2015). *Gba2* knockout mice exhibit gait abnormalities (Woeste et al., 2019) and fertility impairment due to abnormal sperm morphology (Raju et al., 2015). This is consistent with male infertility reported in SPG46 patients (Martin et al., 2013). Lipidomic analysis in Sertoli cells confirmed that loss of GBA2 promotes accumulation of glucosylceramide (Raju et al., 2015). In cerebellum, no difference was observed in the total levels of glucosylceramide between control and *Gba2* knockout mice. However, levels of the gangliosides GM1a, GT1b, GD1b, and GM3 were slightly increased in the cerebellum of *Gba2* knockout mice compared to wild-type mice (Woeste et al., 2019). Yet, the link between accumulation of glucosylceramide and neurodegeneration is not known. It was proposed that accumulation of glucosylceramide could alter the membrane fluidity, which could lead to increased actin polymerization (Raju et al., 2015).

### Alteration of Fatty Acid Metabolism in Syndromes Presenting Spasticity

Several rare neurodegenerative diseases presenting spasticity are implicated in the metabolism of fatty acids. Fatty acids are building blocks for other lipids such as phospholipids, ceramide, cholesterol esters, and thus change in fatty acid metabolism can have an impact on many classes of lipids as illustrated below.

X-linked adrenoleukodystrophy (X-ALD) is an X-linked syndrome caused by mutations in the *ABCD1* gene that codes for the adrenoleukodystrophy protein (ALDP) (Mosser et al., 1993). First, symptoms may occur from childhood to adulthood, leading to progressive demyelination of the central and peripheral nervous system. The earlier the illness begins, the more severe it will be and childhood forms of X-ALD are characterized by a devastating cerebral demyelination, leading to rapid cognitive and motor decline, vegetative state within a few months, and premature death. The clinical spectrum in males with X-ALD ranges from childhood cerebral ALD (CALD: onset 5–12 years, 35–40% of affected males) to slowly progressive spastic paraplegia in adulthood (adrenomyeloneuropathy: onset 20–30 years, 60% of affected males) (Kemp et al., 2012). Approximately 20% of males with adrenomyeloneuropathy will also develop CALD later in life with the same poor prognosis as in children. The only available therapy for CALD is allogenic HSCT (Raymond et al., 2019; Waldhüter et al., 2019). If performed in patients with minimal brain lesions and no/minor clinical symptoms, it may arrest the progression of demyelinating lesions. To overcome the limitations of allogeneic transplantation (lack of donor, graft versus host reaction, high mortality rate), autologous transplantation of HSC corrected *ex vivo* with a lentiviral vector expressing the *ABCD1* gene is currently under clinical trial in children with CALD with very encouraging results (Eichler et al., 2017). Women carriers of *ABCD1* mutations may develop milder forms of the disease, usually with spastic paraplegia, and sometimes peripheral neuropathy, in adulthood (Huffnagel et al., 2019). However, it is increasingly recognized that *ABCD1*-mutated women develop a wide spectrum of neurological diseases with age (Engelen et al., 2014). All male patients present elevated plasma levels of very long chain fatty acids (VLCFA) but they may be normal in female carriers. The accumulation of VLCFA is also detected in lipid molecules or proteins complexing fatty acids. The myelin of X-ALD patients showed a strong enrichment in VLCFA complexed in cholesterol esters and sphingomyelin (Brown et al., 1983), an increased proportion of proteolipid protein (PLP) complexed to monounsaturated VLCFA (Bizzozero et al., 1991), and increased amount of VLCFA in glycerophospholipids (Theda et al., 1992). VLCFA accumulation is also associated with clinically significant microglial activation (Eichler et al., 2008). These observations highlight the variety of effects that can result from the accumulation of VLCFA. This accumulation in X-ALD patients is the consequence of the impaired β-oxidation of VLCFA by peroxisomes. The *ABCD1* product, ALDP, is a membrane transporter of the ATP-binding cassette family of proteins and is localized in peroxisomes (Watkins et al., 1995). It was shown that ALDP can transport the VLCFA from the cytosol across the peroxisome membrane, allowing their β-oxidation (van Roermund et al., 2008). VLCFA accumulation in X-ALD patients is the consequence of the absence of functional ALDP protein.

VLCFA can be obtained from food, but they can also be synthesized in the ER by elongases that are enzymes allowing the elongation of fatty acids (Jakobsson et al., 2006). Heterozygous mutations in the gene encoding the elongase ELOVL4 was associated with autosomal dominant juvenile macular degeneration (Zhang et al., 2001), or spinocerebellar ataxia SCA34 (Ozaki et al., 2015). However, recessive mutations in *ELOVL4* were found in patients presenting ichthyosis, seizures, mental retardation, and spastic paraplegia (Aldahmesh et al., 2011). Deficiency in ELOVL4 in mice led to perinatal death and was associated with a reduction in VLCFA (Cameron et al., 2007), as well as decreased levels of ceramide with omega-hydroxy VLCFA (Li et al., 2007). Recently, a dominant mutation in *ELOVL1* was found in two pediatric patients presenting ichthyosis, spasticity, mild hypomyelination detected on brain MRI, and dysmorphic features (Kutkowska-Kaźmierczak et al., 2018). Expression of the pathogenic variant in cell lines reduced the production of VLCFA (Kutkowska-Kaźmierczak et al., 2018). Analysis of patient cells also revealed a moderate decrease in the levels of ceramide with fatty acids longer than 24 carbons (Mueller et al., 2019). Consistently, knockout of *Elovl1* in mice resulted in decreased levels of ceramide with VLCFA (Sassa et al., 2013). The X-ALD and elongase-linked syndromes highlight the importance of the balance between synthesis and degradation of VLCFA for proper brain function. The interplay between the two cellular function was also demonstrated by the beneficial action of downregulating ELOVL1 in fibroblasts of X-ALD patients (Ofman et al., 2010).

Sjogren Larsson syndrome (SLS) is an autosomal recessive disorder characterized by ichthyosis, spastic paraplegia, and intellectual disability (Sjogren and Larsson, 1957). This syndrome results from the accumulation of fatty aldehydes (Rizzo and Craft, 1991). Consistently, mutations in *ALDH3A2* that encodes a fatty aldehyde dehydrogenase were identified as responsible for SLS (Laurenzi et al., 1996). It was demonstrated that fibroblasts of SLS patients are more sensitive to cell death induced by fatty aldehydes (James and Zoeller, 1997). This effect could be due to reactions between accumulating fatty aldehydes and proteins or lipids such as phosphatidylethanolamine that would disrupt their biochemical functions (James and Zoeller, 1997).

Mutations in genes encoding enzymes involved in the metabolism of various lipid molecules highlight the role of lipids in the physiopathology of HSP. It is important to note that both synthesis and degradation of most classes of lipids are responsible for diseases with overlapping presentation. This highlights the importance of lipid homeostasis for the normal function of polarized cells like neurons or oligodendrocytes. However, it is not clear how the change in lipid metabolism can lead to axon loss or neurodegeneration. Some functional links have been established from the study of other forms of HSP, as discussed in the next part of this review.

## Functional Consequences of the Alteration of Lipid Metabolism

Lipids being major membrane constituents, alteration of their metabolism is likely to alter various membrane-related functions. We will see that alterations of lipid metabolism can affect various cellular functions (Table 2), which could contribute to neurodegeneration. However, it has also emerged in the last few years that mutations in genes affecting various subcellular compartments can secondarily lead to alteration of lipid metabolism, reinforcing its implication in the physiopathology of HSP.

**TABLE 2:** Genes responsible for HSP encoding proteins modifying cellular lipid metabolism.

**Gene**	**Protein name**	**Function**
**Lipid droplets (LD)**		
*ATL* (*SPG3*)	Atlastin	Regulation of LD size
*SPAST* (SPG4)	Spastin	Regulation of LD size and number
*REEP1* (SPG31)	REEP1	Regulation of LD size
*CPT1C* (SPG73)	Carnitine palmitoyl transferase 1C	Regulation of LD size and number
*Spartin* (SPG20)	Spartin	Regulation of LD size and number
*DDHD2* (SPG54)	DDHD2	Triacylglycerol lipase, hydrolysis of LD
**Mitochondrial function**		
*DDHD1 (SPG28)*	DDHD1	Mitochondria fusion/fission, ROS production, ATP synthesis
*DDHD2* (SPG54)	DDHD2	Mitochondria fusion/fission, ROS production, ATP synthesis, regulate levels of cardiolipins
*SERAC1*	SERAC1	Altered levels of cardiolipins, altered function of the respiratory chain
*Spartin* (SPG20)	Spartin	Regulation of mitochondrial membrane potential
*ABCD1* (X-ALD)	Adrenoleukodystrophy protein (ADLP)	Mitochondrial depolarization, ROS production
**Lysosome function**		
*GALC (Krabbe)*	Galactosylcerebrosidase	Accumulation of psychosine
*ARSA (MLD)*	Arylsulfatase A	Accumulation of sulfatides
*ATP13A2* (SPG78)	P5-type ATPase	Regulates lysosomal degradative activity, accumulation of membranes in lysosomes
*AP5Z1* (SPG48)	Subunit ζ of adapter complex 5	Accumulation of membranes in lysosomes
*SPG11*	Spatacsin	Regulation of autophagic lysosome recovery, accumulation of gangliosides in lysosomes
*ZFYVE26* (SPG15)	Spastizin	Regulation of autophagic lysosome recovery, accumulation of membranes in lysosomes
*SERAC1*	SERAC1	Reduced levels of bis-monoacylglycerol-phosphate in lysosomes
***Synaptic function***		
*PLA2G6*	Group VI phospholipase A2, iPLA2b	Alteration of synaptic structure
*PNPLA6/NTE* (SPG39)	Patatin-like phospholipase domain containing 6/Neuropathy target esterase	Reduction of the activity of the secretory pathway
*BSCL2 (SPG17)*	Seipin (N88S variant: altered glycosylation of seipin)	Decreased number of docked synaptic vesicles in sypases
**Myelin/axon maintenance**		
*PLP1* (SPG2)	Proteolipid protein	Constituent of myelin, required for long term axon maintenance
*MAG* (SPG75)	Myelin associated glycoprotein	Constituent of myelin, binds to gangliosides present in axon membrane, required for long term axon maintenance
*GJC2* (SPG44)	Connexin 47	Gap junction protein, required for glial coupling in white matter
*FA2H* (SPG35)	Fatty acid-2 hydroxylase	Decreased levels of galactosylceramide, required for long-term axon maintenance
*GALC* (Krabbe)	Galactosylcerebrosidase	Defective myelin formation and later on demyelination; psychosine-induced toxicity of oligodendrocytes
*ARSA* (MLD)	Arylsulfatase A	Demyelination

### Lipid Droplets

Lipid droplets (LD) are storage organelles that are formed by a core of neutral lipids surrounded by a monolayer of phospholipids. The main lipids stored in LDs are triacylglycerols (TAG) and esterified cholesterol. LDs formation occurs in the membrane of the ER. They are dynamic organelles, playing a key role in lipid metabolism and energy homeostasis (Olzmann and Carvalho, 2019).

Consistent with the formation of LD in the ER membrane, several proteins implicated in the morphogenesis of the ER network have been associated with LD biogenesis or have been shown to modify their dynamics. When overexpressed, atlastin and REEP1 are localized at the rim of LD, and the coexpression of both proteins increases the size of LD (Klemm et al., 2013). Similarly, M1 spastin, but not M87 spastin, was colocalized with LD, and overexpression of the M1 spastin isoform increased the size of LD in HeLa cells, as well as in *Drosophila* fat body, muscle, and nerve tissues (Papadopoulos et al., 2015). Conversely, downregulation of spastin in *Drosophila* or *Caenorhabditis elegans* decreased the number of LD and TAG levels (Papadopoulos et al., 2015). Atlastin loss of function in *C. elegans* or *Drosophila* reduced LD size and TAG levels (Klemm et al., 2013). *Reep1* knockout mice also presented with a lipoatrophy, associated with reduced levels of serum cholesterol and TAG. The number of LD was also reduced in neurons of *Reep1* knockout mice (Renvoisé et al., 2016). Since atlastin-1, spastin, and Reep1 are ER-shaping proteins (Park et al., 2010), these studies nicely show a correlation between ER morphology and accumulation of LD. CPT1C, an atlastin binding protein, has not been shown to alter ER morphology, but its knockout in mouse also decreased the number and size of LD (Rinaldi et al., 2015). It is noteworthy that SPG3 (atlastin-1), SPG4 (spastin), SPG31 (REEP1), and SPG73 (CPT1C) are rather pure forms of HSP. Yet, it is not known whether the alteration of ER morphology or the alteration of LD metabolism is responsible for patients’ phenotype. Recently, it was proposed that M1 spastin promotes the docking of LD to peroxisomes, allowing the transfer of fatty acids from LD to peroxisomes. Expression of a mutant spastin prevented this transfer and led to accumulation of peroxided lipids, which could contribute to cell death (Chang et al., 2019).

Spartin and seipin, which are mutated in the complex forms of HSP SPG20 and SPG17, respectively, were also found in contact with LD (Szymanski et al., 2007; Eastman et al., 2009). Spartin interacts with the E3 ubiquitin ligases AIP4 and AIP5 (Edwards et al., 2009), and it was proposed to promote the ubiquitylation of lipid droplet proteins (Hooper et al., 2010). Downregulation of spartin leads to increased LD number and size (Eastman et al., 2009). Seipin is also implicated in the regulation of ER-LD contact and loss of seipin function impaired the formation of LD in fibroblasts (Szymanski et al., 2007; Salo et al., 2016). However, SPG17 is due to gain-of-function mutation in the *BSCL2* gene, altering glycosylation sites of the protein, and the consequences of these mutations on LD metabolism have not yet been investigated.

Another complex form of HSP associated with the accumulation of LD is SPG54. LD accumulated prominently in neurons when the main brain TAG lipase, DDHD2, is knocked out (Inloes et al., 2014). The loss of Ddhd2 in mice was also responsible for motor and cognitive impairment. In this mouse model, LD accumulated in neurons, including axons and dendrites (Inloes et al., 2014). It was proposed that accumulation of LD in neuronal processes can lead to swellings and disturb intraneuronal trafficking, which could contribute to neurodegeneration. *Ddhd2* knockdown in *Drosophila* reduced the number of active zones at synaptic terminals (Schuurs-Hoeijmakers et al., 2012). LD may thus disrupt trafficking of proteins and lipids required for synapse assembly (Pennetta and Welte, 2018).

Interestingly, these studies suggest that mutations leading to reduced LD are associated with rather pure forms of HSP. In contrast, other forms of HSP that were associated with accumulation of LD are rather complex HSP. However, it is not clear whether the severity of the phenotype is only due to alteration of LD. Indeed, LD are in interaction with other subcellular compartments such as mitochondria or lysosomes that can also be affected and could contribute to the physiopathology of HSP.

### Mitochondria Dysfunction

Mitochondria play a key role in lipid metabolism, as they can degrade lipids through β-oxidation, or contribute to synthesis of TAG (Benador et al., 2019). It is interesting to note that loss of DDHD2 or spartin that are associated with accumulation of LD also leads to mitochondrial dysfunction (Joshi and Bakowska, 2011; Maruyama et al., 2018). Yet, neurons are not known to utilize lipids as an energy source for mitochondria, raising the question of other possible roles of lipids in the regulation of mitochondrial function. Alternatively, motor neuron dysfunction could also result from altered energy supply from glial cells that use lipids as energetic substrates.

DDHD1 and DDHD2 were initially described as phosphatidic acid (PA)-preferring phospholipases A1 (Higgs et al., 1998; Inoue et al., 2012). The content of PA in outer mitochondrial membrane can regulate the dynamics of mitochondria fusion and fission (Kameoka et al., 2018). Knockdown of *DDHD1* or *DDHD2* in some cells types, but not all of them, promoted elongation of mitochondria, probably by enhancing their fusion (Baba et al., 2014). The equilibrium between fusion and fission of mitochondria is required to clear damaged mitochondria (Twig et al., 2008). Loss of DDHD2 in mouse embryonic fibroblasts increased mitochondrial membrane potential and reduced ATP production and O_2_ consumption. These mitochondrial dysfunction were associated with production of reactive oxygen species (Maruyama et al., 2018). Similar to loss of DDHD2, lymphoblasts of SPG28 patients devoid of DDHD1 also presented higher ROS production and lower ATP production compared to control cells (Tesson et al., 2012). Excessive fusion observed in the absence of DDHD1 or DDHD2 could explain the accumulation of dysfunctional mitochondria and thus impaired ATP production, which could contribute to neurodegeneration.

Importantly, the alteration of mitochondrial function in *DDHD2* knockout fibroblasts was not associated with the accumulation of LD or increased levels of TAG, but it was associated with decreased levels of cardiolipin, a mitochondria-specific lipid class (Maruyama et al., 2018). Cardiolipin can regulate the fusion of mitochondria and the formation of mitochondrial respiratory supercomplexes (Mileykovskaya and Dowhan, 2014; Kameoka et al., 2018). In fibroblasts of patients with *SERAC1* mutations (MEGDEL syndrome), the levels of cardiolipin were increased compared to controls. This was associated with a dysfunction of the mitochondrial respiratory chain (Wortmann et al., 2012). It has not been investigated whether mitochondrial function was altered in fibroblasts of HSP patients with *SERAC1* mutations. Spartin, implicated in SPG20, was also shown to interact with cardiolipins and was present on the outer mitochondrial membrane. Downregulation of spartin decreased mitochondrial membrane potential (Joshi and Bakowska, 2011), but it is not clear whether this effect is caused by a change in the levels of mitochondria lipid composition.

*In vitro* experiments showed that exposition to VLCFA induced death of astrocytes and oligodendrocytes (Hein et al., 2008). VLCFA exposition deregulated intracellular calcium homeostasis in neurons, astrocytes, and oligodendrocytes, but the latter were the most affected cell type (Hein et al., 2008). VLCFA exposition induced mitochondrial depolarization and reactive oxygen species production (Hein et al., 2008), which could contribute to oligodendrocyte death. Consistently, astrocytes derived from *Abcd1* knockout mice exhibited impaired energy metabolism and increased ROS production when exposed to supraphysiological concentration of VLCFA (Kruska et al., 2015).

Change in mitochondrial function was also observed in fibroblasts and lymphoblasts from SPG49/56 patients. In the absence of functional CYP21U, the oxygen consumption was reduced and was associated with increased oxidative stress (Tesson et al., 2012). The subcellular localization of CYP2U1 is still not clear. This cytochrome was proposed to metabolize arachidonic acid into 19- and 20-HETE (Chuang et al., 2004), or to oxidize the endocannabinoid N-arachidonoylserotonin to downregulate its action (Siller et al., 2014). However, it is not known whether these molecules can regulate mitochondrial function directly or whether this is mediated by other metabolites, not yet identified, of this cytochrome P450.

### Lysosomal Dysfunction

The clearest link between alteration of lysosome function and spasticity is provided by Krabbe and metachromatic leukodystrophy patients. Indeed, these two pathologies belong to the group of lysosomal storage disorders. Both diseases result from the loss of activity of a lysosomal enzyme leading to abnormal accumulation of substrates: psychosine (Krabbe) and sulfatides (Metachromatic leukodystrophy). Why such accumulation of substrates leads to spasticity is not clear.

Several other forms of HSP have also been proposed to be associated with impaired lysosomal function. Mutations of *ATP13A2* were found in families with complex hereditary spastic paraplegia (SPG78) (Estrada-Cuzcano et al., 2017). ATP13A2 is a P5ATPase that is mainly localized in the membrane of lysosomes (Ramirez et al., 2006), and dysfunction of this protein was shown to be responsible for impaired lysosomal degradation (Dehay et al., 2012). Consistently, homozygous mutations in fibroblasts of SPG78 patients increased the number of lysosomes and impaired lysosomal degradative activity, and electron microscopy analysis revealed that lysosomes accumulated abnormal material consisting of whirls and stacks of membranes (Estrada-Cuzcano et al., 2017). This was reminiscent of neuronal ceroid lipofuscinosis, also found in patients mutated in this gene (Bras et al., 2012). Similar accumulation of lysosomal membranes was also observed in fibroblasts of SPG48 patients with loss-of-function mutation in the ζ subunit of the AP-5 complex (Hirst et al., 2015). Electron microscopy analysis of fibroblasts of SPG15 patients and neurons of *Spg15* knockout mice showed the accumulation of zebra or fingerprint bodies (Khundadze et al., 2013; Renvoisé et al., 2014), which are lysosomes with accumulation of membranes similar to those found in some lysosomal storage disorders (Parkinson-Lawrence et al., 2010). Analysis of SPG11 patient fibroblasts, in contrast, did not reveal accumulation of lamellar structures by electron microscopy. However, in brains of *Spg15*, *Spg11*, and *Spg48* knockout mice, ultrastructural analysis showed the presence of electron dense deposits that looked like lipofuscin (Khundadze et al., 2013, 2019; Varga et al., 2015; Branchu et al., 2017). In all three models, the presence of these deposits was proposed to result from the accumulation of autolysosomes or impaired autophagic clearance. In the case of *Spg11* knockout mice, the lysosomal deposits were shown to contain lipids, and a lipidomic analysis revealed that the lysosomes accumulated simple gangliosides (Boutry et al., 2018). Whether gangliosides accumulate upon loss of function of spastizin or AP-5ζ is not known. Likewise, it is not clear why gangliosides accumulate in absence of spatacsin, the product of *SPG11*. It was proposed that such accumulation could be a consequence of impaired lysosomal membrane recycling (Boutry et al., 2018), but other mechanisms could contribute to this accumulation. For example, the levels of bis-monoacylglycerol-phosphate (BMP), which facilitates the degradation of GM2 ganglioside (Anheuser et al., 2015), were reduced in fibroblasts of patients with *SERAC1* mutations compared to controls (Wortmann et al., 2012). Importantly, mutations in *SPG11*, *SPG15*, *SPG48*, *ATP13A2*, and *SERAC1* have been associated with parkinsonism in some patients (Ramirez et al., 2006; Anheim et al., 2009; Schicks et al., 2011; Hirst et al., 2016; Ma et al., 2018). The clinical overlap between these patients, as well as the similarities observed in the lysosomal dysfunction suggest that these HSP entities may share some physiopathological pathways, although some differences may exist between them (Vantaggiato et al., 2019). Accumulation of membrane structures in lysosomes has also been observed in cells derived from *Spg4* or *Spg31* knockout mice. These defects resulted from impaired ER-mediated endosomal tubule fission (Allison et al., 2017). Yet, it is not clear whether these lysosomal structures share similarities in composition with the lysosomes accumulating membranes observed in fibroblasts of SPG11, SPG15, SPG48, or ATP13A2-deficient patients.

In SPG11 models, decreased gangliosides synthesis prevented neuronal death *in vitro* and improved the motor phenotype in a zebrafish model, suggesting that accumulation of gangliosides could contribute to neurodegeneration (Boutry et al., 2018). These results were notably obtained using miglustat, a non-specific inhibitor of glucosylceramide synthase that is used in the treatment of Gaucher disease. However, miglustat poorly crosses the blood–brain barrier and is not efficient for symptoms of neuropathic Gaucher Disease (Schiffmann et al., 2008). Furthermore, in a mouse model of Sandhoff disease, miglustat increased the levels of brain glycosphingolipids (Ashe et al., 2011), questioning its efficacy in the central nervous system. Decreasing ganglioside synthesis is thus of therapeutic interest for SPG11 patients, but it is still unsure whether miglustat is a good therapeutic option for these patients. Further studies are thus required to validate this therapeutic strategy. If such a therapy was efficient in SPG11, it would be worth validating whether a similar strategy could also be efficient in other forms of HSP presenting alteration of lysosomal function and a phenotypic presentation overlapping with SPG11 patients. Besides synthesis of gangliosides, other studies have shown a beneficial effect of clinically used GSK3 blocker tideglusib on the neurodevelopmental phenotype observed in SPG11 models (Mishra et al., 2016; Pérez-Brangulí et al., 2019). However, it is not known yet whether such a treatment would affect the metabolism of gangliosides or act on a different pathway. It has also not been evaluated whether restoration of normal development in SPG11 could prevent neurodegeneration that occurs later in life of SPG11 patients.

### Alteration of Synaptic Function

Neuropathological investigations on autopsic cases of HSP have proposed a “dying back” mechanism of axonal degeneration (DeLuca et al., 2004). Such mechanisms would be compatible with a primary alteration of synaptic function. Exocytosis and endocytosis of synaptic vesicles have been thought to be mainly mediated by proteins, but it has emerged that lipid composition of membranes and lipid mediators also play a key role in these processes (Darios et al., 2007; Mochel, 2018). It is therefore tempting to speculate that alteration of lipid metabolism observed in some HSP would impair synaptic function and underlie some symptoms of the disease. For example, the product of *PLA2G6*, the calcium-independent phospholipase A2 β (iPLA2β), is localized in axon terminals and dendritic spines of neurons (Ong et al., 2005) where it releases polyunsaturated fatty acids (PUFAs) from brain phospholipids (Green et al., 2008). Accordingly, brains of *Pla2g6* knockout mice present alteration of some PUFAs (Beck et al., 2011). Ultrastructural analyses of the posterior horn in this model showed the presence of loose presynaptic membranes containing synaptic vesicles (Beck et al., 2011). This insufficient membrane remodeling was proposed to contribute to the generation of axonal spheroids (Beck et al., 2011). Synaptic dysfunction could also underlie early behavioral symptoms in these mice, before the onset of neurodegeneration. Similarly, deficiency in NTE, another phospholipase associated with HSP, is associated with increased levels of phosphatidylcholine in brain—as described in the first part of the review—and a 20% decrease of protein secretion (Read et al., 2009). Alteration of the secretory pathway could affect synaptic vesicles and contribute to neuronal dysfunction long before neurodegeneration. Yet, iPLA2β and NTE have different lipid targets and may act by different mechanisms to regulate synaptic function. DDHD2, which was proposed to have a PLA1 action, also leads to synaptic defects in a drosophila model (Schuurs-Hoeijmakers et al., 2012). It would be interesting to further analyze how the loss of function of these enzymes contributes to synaptic dysfunction and underlie some early symptoms of HSP.

Alteration of synaptic function has also been observed upon expression of N88S seipin mutant in neurons, which decreased the number of synaptic vesicles docked to the plasma membrane. This alteration decreased the amplitude of evoked postsynaptic response (Wei et al., 2014). Yet, the link between the function of seipin in LD and the alteration of synaptic vesicles docking is unknown. Similarly, loss of spastin or atlastin, which regulates LD formation as illustrated above, affects synaptic functions (Trotta et al., 2004; De Gregorio et al., 2017). Whether this is related to the change in ER morphology due to loss of spastin or atlastin, or to the action of these proteins on LD remains to be established.

### Importance of Myelin for Axon Maintenance

The most prominent lipid component of the brain is myelin that surrounds axons and promotes fast propagation of membrane depolarization. Most, if not all, forms of complex HSP are associated with white matter abnormalities detected on brain MRI. On the other hand, most, if not all, forms of leukodystrophies can present with spastic paraplegia such as Krabbe disease and metachromatic leukodystrophy. Likewise, it becomes increasingly difficult to make a distinction between HSP and leukodystrophy genes as illustrated by FA2H (Kruer et al., 2010).

Mutations in proteolipid protein gene (*PLP1*), *GJC2*, and *MAG* are responsible for a variety of phenotype ranging from hereditary spastic paraplegias (SPG2, SPG44, and SPG75, respectively) to neurodevelopmental disorders including Pelizaeus-Merzbacher disease, a hypomyelinating disorder of the central nervous system (Saugier-Veber et al., 1994; Orthmann-Murphy et al., 2009; Novarino et al., 2014; Lossos et al., 2015). The proteins encoded by these genes are essential for the maintenance of the myelin and axon integrity. Furthermore, loss of PLP1 in an oligodendrocyte cell line led to decreased levels of ethanolamine plasmalogen (Wood et al., 2011), highlighting the link between lipid metabolism and myelin maintenance. *PLP1* encodes two proteins, PLP and DM20, that are essential constituents of myelin. An autopsic case of SPG2 showed widespread white matter pallor in the central nervous system. Demyelination was associated with loss of axons and remaining fibers contained spheroids (Suzuki et al., 2011). Similarly, *Plp1* knockout mice assembled compact myelin sheaths, but subsequently showed progressive degeneration of axons (Griffiths, 1998), suggesting that maintenance of myelin is required to sustain neuronal function. This was confirmed by the specific deletion of *Plp1* in oligodendroglial lineage, demonstrating that PLP is essential in oligodendrocytes to sustain axonal function and prevent axonal degeneration (Lüders et al., 2017). Loss of PLP was shown to alter fast axonal transport, leading to accumulation of membranous organelles and formation of axonal swellings (Edgar et al., 2004).

Similarly to loss of Plp, *Mag* knockout mice did not show impaired myelination, but when they became older than 8 months, they presented alteration of the maintenance of axon-myelin units, resulting in both axon and myelin degeneration (Fruttiger et al., 1995). MAG is expressed on the myelin membrane wrap that is directly apposed to the membrane of neurons, and it binds to receptors on axons (Schnaar and Lopez, 2009). Among the putative receptors on axon membranes, GD1a and GT1b were identified as potent MAG receptors (Yang et al., 1996). *B4galnt1* knockout mice, which are deficient in a key enzyme for ganglioside extension, lack GD1a and GT1b and present progressive axon degeneration (Sheikh et al., 1999; Chiavegatto et al., 2000). Expression of B4GALNT1 specifically in neurons in the *B4galnt1^–/–^* mice prevented axon degeneration, suggesting that neuronal gangliosides are critical to maintain axon integrity (Yao et al., 2014). Interestingly, comparison of *Mag*, *B4galnt1*, and double knockout mice in the same genetic background showed similar axon degeneration, suggesting that oligodendroglial MAG binding to neuronal gangliosides contributes to maintain axon function (Pan et al., 2005).

Beside mutations affecting proteins that are myelin constituents, other mutations affect the lipid composition of myelin. Myelin is highly enriched in sphingolipids containing 2-hydroxylated fatty acids that require the FA2H enzyme for their synthesis, as illustrated in the first section of the review. The absence of FA2H in mouse did not prevent the formation of myelin sheaths. However, as mice were aging, axon and myelin sheath degeneration was observed (Zoller et al., 2008). Similar to the observations made in mice devoid of PLP or MAG, this suggests that axon degeneration is a consequence of impaired support provided by myelin. Specific deletion of FA2H in Schwann cells and oligodendrocytes showed demyelination similar to that observed in a constitutive *Fa2h* knockout mouse model (Potter et al., 2011). However, these mice did not show impaired cognitive function as observed in constitutive knockout, suggesting that FA2H could also have important function outside myelinating cells (Potter et al., 2011). Levels of 2-hydroxygalactosylceramide are also strongly reduced in the *Aldh3a2* knockout mouse model of Sjorgren–Larsson syndrome, due to inactivation of FA2H (Kanetake et al., 2019). Consistent with the *Fa2h* knockout, the formation of myelin was not impaired in *Aldh3a2* knockout mice (Kanetake et al., 2019).

As already discussed, X-ALD patients present progressive demyelination. In these patients and in a mouse model of the disease, the composition of myelin is altered (Brown et al., 1983; Bizzozero et al., 1991; Theda et al., 1992; Hama et al., 2018), but it is still not known whether this change in lipid composition contributes to demyelination. Patients affected by Krabbe disease or metachromatic leukodystrophy also present a strong demyelination, although the molecular mechanism underlying this phenomenon is still unclear. Loss of arylsulfatase A activity both in metachromatic leukodystrophy patients and in a mouse model of the disease leads to a strong accumulation of sulfatides, a class of lipids enriched in myelin. Yet, the mouse model of this disease did not present any gross white matter defect (Hess et al., 1996). In Krabbe disease, defective myelin formation as well as demyelination at a late disease stage have been observed in the natural Twitcher mouse model of the disease (Takahashi and Suzuki, 1984; Inamura et al., 2018). The alterations were proposed to result from impaired differentiation and survival of oligodendrocytes, due to accumulation of psychosine (Inamura et al., 2018).

Abnormal white matter has been detected on brain MRI in many HSP subtypes. Therefore, the loss of support provided by myelin to maintain axon function could contribute to the physiopathology in many forms of HSP. However, the mechanism of myelin loss in many forms of HSP is not known, and it will be important to determine whether loss of myelin is the primary defect or whether it is secondary or concomitant to axon degeneration.

## Conclusion

HSP can be due to various mutations in genes encoding proteins involved in lipid metabolism, affecting most classes of lipids (sterols, fatty acids, phospholipids, and sphingolipids). This review of the literature highlights that alteration of both synthesis and degradation of the various classes of lipids can lead to HSP or HSP-related disorders, highlighting the importance of lipid homeostasis for the brain physiology. Based on current knowledge, the products of some genes can be connected within metabolic pathways, but many gaps exist between these products. Since the genetic cause is still unknown in about 50% of HSP patients, the identification of new causative genes may help to fill this gap. However, the identification of genes responsible for diseases has become challenging, as the main genes have likely been identified. Combination of genomic with lipidomic analysis could help us to uncover new genes and complete the map of genes responsible for HSP that are implicated in lipid metabolism.

As illustrated here, various steps of the metabolism of lipid molecules can be affected, leading to alteration in various subcellular compartments. Alteration of mitochondrial functions, oxidative stress or impairment of axonal transport can occur in some forms of HSP associated with alteration of lipid metabolism. Yet, the link between alteration of lipid metabolism and neurodegeneration is still unclear in most forms of HSP. Indeed, many studies have been performed in cell lines or cells derived from patients to demonstrate which lipid class was primarily affected. As shown by the investigation of myelin–axon interaction, studies modulating lipid metabolism in animal models, or at least cultured neuronal models, are now required to evaluate the role of lipids in neurodegeneration in HSP. This kind of study will be critical to develop therapeutic strategies. Indeed, metabolic processes, including lipid metabolism, are in theory druggable and understanding the role of lipids in neurodegeneration could help to identify therapeutic targets.

## Author Contributions

All authors listed have made a substantial, direct and intellectual contribution to the work, and approved it for publication.

## Conflict of Interest

The authors declare that the research was conducted in the absence of any commercial or financial relationships that could be construed as a potential conflict of interest.

## References

[B1] Aharon-Peretz J. Rosenbaum H. Gershoni-Baruch R. (2004). Mutations in the glucocerebrosidase gene and Parkinson’s disease in ashkenazi jews. *N. Engl. J. Med.* 351 1972–1977. 10.1056/NEJMoa033277 15525722

[B2] Ahmed M. Y. Al-Khayat A. Al-Murshedi F. Al-Futaisi A. Chioza B. A. Pedro Fernandez-Murray J. (2017). A mutation of *EPT1 (SELENOI)* underlies a new disorder of Kennedy pathway phospholipid biosynthesis. *Brain* 140 547–554. 10.1093/brain/aww318 28052917 PMC5382949

[B3] Alazami A. M. Adly N. Al Dhalaan H. Alkuraya F. S. (2011). A nullimorphic ERLIN2 mutation defines a complicated hereditary spastic paraplegia locus (SPG18) *Neurogenetics* 12 333–336. 10.1007/s10048-011-02918 21796390 PMC3215864

[B4] Aldahmesh M. A. Mohamed J. Y. Alkuraya H. S. Verma I. C. Puri R. D. Alaiya A. A. (2011). Recessive mutations in ELOVL4 cause ichthyosis, intellectual disability, and spastic quadriplegia. *Am. J. Hum. Genet.* 89 745–750. 10.1016/j.ajhg.2011.10.011 22100072 PMC3234380

[B5] Alderson N. L. Rembiesa B. M. Walla M. D. Bielawska A. Bielawski J. Hama H. (2004). The human *FA2H* gene encodes a fatty acid 2-hydroxylase. *J. Biol. Chem.* 279 48562–48568. 10.1074/jbc.M406649200 15337768

[B6] Allison R. Edgar J. R. Pearson G. Rizo T. Newton T. Günther S. (2017). Defects in ER–endosome contacts impact lysosome function in hereditary spastic paraplegia. *J. Cell Biol.* 216 1337–1355. 10.1083/jcb.201609033 28389476 PMC5412567

[B7] Amador M. D. M. Masingue M. Debs R. Lamari F. Perlbarg V. Roze E. (2018). Treatment with chenodeoxycholic acid in cerebrotendinous xanthomatosis: clinical, neurophysiological, and quantitative brain structural outcomes. *J. Inherit. Metab. Dis.* 41 799–807. 10.1007/s10545-018-01627 29560583

[B8] Anheim M. Lagier-Tourenne C. Stevanin G. Fleury M. Durr A. Namer I. J. (2009). SPG11 spastic paraplegia. A new cause of juvenile parkinsonism. *J. Neurol.* 256 104–108. 10.1007/s00415-009-0083-3 19224311

[B9] Anheuser S. Breiden B. Schwarzmann G. Sandhoff K. (2015). Membrane lipids regulate ganglioside GM2 catabolism and GM2 activator protein activity. *J. Lipid Res.* 56 1747–1761. 10.1194/jlr.M061036 26175473 PMC4548779

[B10] Ashe K. M. Bangari D. Li L. Cabrera-Salazar M. A. Bercury S. D. Nietupski J. B. (2011). Iminosugar-based inhibitors of glucosylceramide synthase increase brain glycosphingolipids and survival in a mouse model of sandhoff disease. *PLoS ONE* 6:e21758. 10.1371/journal.pone.0021758 21738789 PMC3126858

[B11] Baba T. Kashiwagi Y. Arimitsu N. Kogure T. Edo A. Maruyama T. (2014). Phosphatidic acid (PA)-preferring phospholipase A1 regulates mitochondrial dynamics. *J. Biol. Chem.* 289 11497–11511. 10.1074/jbc.M113.531921 24599962 PMC4036285

[B12] Beck G. Sugiura Y. Shinzawa K. Kato S. Setou M. Tsujimoto Y. (2011). Neuroaxonal dystrophy in calcium-independent phospholipase A2 deficiency results from insufficient remodeling and degeneration of mitochondrial and presynaptic membranes. *J. Neurosci.* 31 11411–11420. 10.1523/JNEUROSCI.0345-11.2011 21813701 PMC6623367

[B13] Benador I. Y. Veliova M. Liesa M. Shirihai O. S. (2019). Mitochondria bound to lipid droplets: where mitochondrial dynamics regulate lipid storage and utilization. *Cell Metab.* 29 827–835. 10.1016/j.cmet.2019.02.011 30905670 PMC6476311

[B14] Berginer V. M. Salen G. Shefer S. (1984). Long-term treatment of cerebrotendinous xanthomatosis with chenodeoxycholic acid. *N. Engl. J. Med.* 311 1649–1652. 10.1056/NEJM198412273112601 6504105

[B15] Bizzozero O. A. Zuñiga G. Lees M. B. (1991). Fatty acid composition of human myelin proteolipid protein in peroxisomal disorders. *J. Neurochem.* 56 872–878. 10.1111/j.1471-4159.1991.tb02003.x 1704424

[B16] Boukhris A. Schule R. Loureiro J. L. Lourenço C. M. Mundwiller E. Gonzalez M. A. (2013). Alteration of ganglioside biosynthesis responsible for complex hereditary spastic paraplegia. *Am. J. Hum. Genet.* 93 118–123. 10.1016/j.ajhg.2013.05.006 23746551 PMC3710753

[B17] Boutry M. Branchu J. Lustremant C. Pujol C. Pernelle J. Matusiak R. (2018). Inhibition of lysosome membrane recycling causes accumulation of gangliosides that contribute to neurodegeneration. *Cell Rep.* 23 3813–3826. 10.1016/j.celrep.2018.05.098 29949766 PMC6045775

[B18] Boutry M. Morais S. Stevanin G. (2019a). Update on the genetics of spastic paraplegias. *Curr. Neurol. Neurosci. Rep.* 19:18 10.1007/s11910-019-09302 30820684

[B19] Boutry M. Pierga A. Matusiak R. Branchu J. Houllegatte M. Ibrahim Y. (2019b). Loss of spatacsin impairs cholesterol trafficking and calcium homeostasis. *Commun. Biol.* 2:380. 10.1038/s42003-019-0615-z 31637311 PMC6797781

[B20] Bowen D. M. Radin N. S. (1968). Hydroxy fatty acid metabolism in brain. *Adv. Lipid Res.* 6 255–272. 10.1016/b978-1-4831-9942-9.50013-9 4180000

[B21] Branchu J. Boutry M. Sourd L. Depp M. Leone C. Corriger A. (2017). Loss of spatacsin function alters lysosomal lipid clearance leading to upper and lower motor neuron degeneration. *Neurobiol. Dis.* 102 21–37. 10.1016/j.nbd.2017.02.007 28237315 PMC5391847

[B22] Bras J. Verloes A. Schneider S. A. Mole S. E. Guerreiro R. J. (2012). Mutation of the parkinsonism gene ATP13A2 causes neuronal ceroid-lipofuscinosis. *Hum. Mol. Genet.* 21 2646–2650. 10.1093/hmg/dds089 22388936 PMC3363329

[B23] Braverman N. E. Moser A. B. (2012). Functions of plasmalogen lipids in health and disease. *Biochim. Biophys. Acta BBA – Mol. Basis Dis.* 1822 1442–1452. 10.1016/j.bbadis.2012.05.008 22627108

[B24] Browman D. T. (2006). Erlin-1 and erlin-2 are novel members of the prohibitin family of proteins that define lipid-raft-like domains of the ER. *J. Cell Sci.* 119 3149–3160. 10.1242/jcs.03060 16835267

[B25] Brown F. R. Chen W. W. Kirschner D. A. Frayer K. L. Powers J. M. Moser A. B. (1983). Myelin membrane from adrenoleukodystrophy brain white matter? Biochemical properties. *J. Neurochem.* 41 341–348. 10.1111/j.1471-4159.1983.tb04748.x 6875541

[B26] Cameron D. J. Tong Z. Yang Z. Kaminoh J. Kamiyah S. Chen H. (2007). Essential role of Elovl4 in very long chain fatty acid synthesis, skin permeability barrier function, and neonatal survival. *Int. J. Biol. Sci.* 3 111–119. 10.7150/ijbs.3.111 17304340 PMC1796949

[B27] Carrasco P. Sahún I. McDonald J. Ramírez S. Jacas J. Gratacós E. (2012). Ceramide levels regulated by carnitine palmitoyltransferase 1c control dendritic spine maturation and cognition. *J. Biol. Chem.* 287 21224–21232. 10.1074/jbc.M111.337493 22539351 PMC3375544

[B28] Casals N. Zammit V. Herrero L. Fadó R. Rodríguez-Rodríguez R. Serra D. (2016). Carnitine palmitoyltransferase 1C: from cognition to cancer. *Prog. Lipid Res.* 61 134–148. 10.1016/j.plipres.2015.11.004 26708865

[B29] Chang C.-L. Weigel A. V. Ioannou M. S. Pasolli H. A. Xu C. S. Peale D. R. (2019). Spastin tethers lipid droplets to peroxisomes and directs fatty acid trafficking through ESCRT-III. *J. Cell Biol.* 218 2583–2599. 10.1083/jcb.201902061 31227594 PMC6683741

[B30] Chang J. Lee S. Blackstone C. (2014). Spastic paraplegia proteins spastizin and spatacsin mediate autophagic lysosome reformation. *J. Clin. Invest.* 124 5249–5262. 10.1172/JCI77598 25365221 PMC4348974

[B31] Chen W.-J. (2019). *PCSK9 Inhibitor Treatment for Patients With Hereditary Spastic Paraplegia Type 5*. Available at: https://clinicaltrials.gov/ct2/show/NCT04101643.

[B32] Chiavegatto S. Sun J. Nelson R. J. Schnaar R. L. (2000). A functional role for complex gangliosides: motor deficits in GM2/GD2 synthase knockout mice. *Exp. Neurol.* 166 227–234. 10.1006/exnr.2000.7504 11085888

[B33] Chuang S. S. Helvig C. Taimi M. Ramshaw H. A. Collop A. H. Amad M. (2004). CYP2U1, a novel human thymus- and brain-specific cytochrome P450, Catalyzes ω- and (ω-1)-Hydroxylation of Fatty Acids. *J. Biol. Chem.* 279 6305–6314. 10.1074/jbc.M311830200 14660610

[B34] Dard R. Meyniel C. Touitou V. Stevanin G. Lamari F. Durr A. (2017). Mutations in DDHD1, encoding a phospholipase A1, is a novel cause of retinopathy and neurodegeneration with brain iron accumulation. *Eur. J. Med. Genet.* 60 639–642. 10.1016/j.ejmg.2017.08.015 28818478

[B35] Darios F. Connell E. Davletov B. (2007). Phospholipases and fatty acid signalling in exocytosis: phospholipases and fatty acid signalling in exocytosis. *J. Physiol.* 585 699–704. 10.1113/jphysiol.2007.136812 17584839 PMC2375517

[B36] De Gregorio C. Delgado R. Ibacache A. Sierralta J. Couve A. (2017). *Drosophila* Atlastin in motor neurons is required for locomotion and presynaptic function. *J. Cell Sci.* 130 3507–3516. 10.1242/jcs.201657 28860117

[B37] Dehay B. Ramirez A. Martinez-Vicente M. Perier C. Canron M.-H. Doudnikoff E. (2012). Loss of P-type ATPase ATP13A2/PARK9 function induces general lysosomal deficiency and leads to Parkinson disease neurodegeneration. *Proc. Natl. Acad. Sci. U.S.A.* 109 9611–9616. 10.1073/pnas.1112368109 22647602 PMC3386132

[B38] DeLuca G. C. Ebers G. C. Esiri M. M. (2004). The extent of axonal loss in the long tracts in hereditary spastic paraplegia. *Neuropathol. Appl. Neurobiol.* 30 576–584. 10.1111/j.1365-2990.2004.00587.x 15540998

[B39] Dietschy J. M. Turley S. D. (2004). Thematic review series: brain lipids. Cholesterol metabolism in the central nervous system during early development and in the mature animal. *J. Lipid Res.* 45 1375–1397. 10.1194/jlr.R400004-JLR200 15254070

[B40] Dodge J. C. Treleaven C. M. Pacheco J. Cooper S. Bao C. Abraham M. (2015). Glycosphingolipids are modulators of disease pathogenesis in amyotrophic lateral sclerosis. *Proc. Natl. Acad. Sci. U.S.A.* 112 8100–8105. 10.1073/pnas.1508767112 26056266 PMC4491749

[B41] Doi H. Ushiyama M. Baba T. Tani K. Shiina M. Ogata K. (2015). Late-onset spastic ataxia phenotype in a patient with a homozygous DDHD2 mutation. *Sci. Rep.* 4:7132. 10.1038/srep07132 25417924 PMC5384088

[B42] Duell P. B. Salen G. Eichler F. S. DeBarber A. E. Connor S. L. Casaday L. (2018). Diagnosis, treatment, and clinical outcomes in 43 cases with cerebrotendinous xanthomatosis. *J. Clin. Lipidol.* 12 1169–1178. 10.1016/j.jacl.2018.06.008 30017468

[B43] Eastman S. W. Yassaee M. Bieniasz P. D. (2009). A role for ubiquitin ligases and Spartin/SPG20 in lipid droplet turnover. *J. Cell Biol.* 184 881–894. 10.1083/jcb.200808041 19307600 PMC2699154

[B44] Edgar J. M. McLaughlin M. Yool D. Zhang S.-C. Fowler J. H. Montague P. (2004). Oligodendroglial modulation of fast axonal transport in a mouse model of hereditary spastic paraplegia. *J. Cell Biol.* 166 121–131. 10.1083/jcb.200312012 15226307 PMC2172145

[B45] Edvardson S. Hama H. Shaag A. Gomori J. M. Berger I. Soffer D. (2008). Mutations in the fatty acid 2-hydroxylase gene are associated with leukodystrophy with spastic paraparesis and dystonia. *Am. J. Hum. Genet.* 83 643–648. 10.1016/j.ajhg.2008.10.010 19068277 PMC2668027

[B46] Edwards T. L. Clowes V. E. Tsang H. T. H. Connell J. W. Sanderson C. M. Luzio J. P. (2009). Endogenous spartin (SPG20) is recruited to endosomes and lipid droplets and interacts with the ubiquitin E3 ligases AIP4 and AIP5. *Biochem. J.* 423 31–39. 10.1042/BJ20082398 19580544 PMC2762690

[B47] Eichler F. Duncan C. Musolino P. L. Orchard P. J. De Oliveira S. Thrasher A. J. (2017). Hematopoietic stem-cell gene therapy for cerebral adrenoleukodystrophy. *N. Engl. J. Med.* 377 1630–1638. 10.1056/NEJMoa1700554 28976817 PMC5708849

[B48] Eichler F. S. Ren J.-Q. Cossoy M. Rietsch A. M. Nagpal S. Moser A. B. (2008). Is microglial apoptosis an early pathogenic change in cerebral X-linked adrenoleukodystrophy? *Ann. Neurol.* 63 729–742. 10.1002/ana.21391 18571777

[B49] Engelen M. Barbier M. Dijkstra I. M. E. Schür R. de Bie R. M. A. Verhamme C. (2014). X-linked adrenoleukodystrophy in women: a cross-sectional cohort study. *Brain* 137 693–706. 10.1093/brain/awt361 24480483

[B50] Estrada-Cuzcano A. Martin S. Chamova T. Synofzik M. Timmann D. Holemans T. (2017). Loss-of-function mutations in the ATP13A2/PARK9 gene cause complicated hereditary spastic paraplegia (SPG78). *Brain* 140 287–305. 10.1093/brain/aww307 28137957 PMC5278306

[B51] Fruttiger M. Montag D. Schachner M. Martini R. (1995). Crucial role for the myelin-associated glycoprotein in the maintenance of axon-myelin integrity. *Eur. J. Neurosci.* 7 511–515. 10.1111/j.1460-9568.1995.tb00347.x 7539694

[B52] Fujita M. Watanabe R. Jaensch N. Romanova-Michaelides M. Satoh T. Kato M. (2011). Sorting of GPI-anchored proteins into ER exit sites by p24 proteins is dependent on remodeled GPI. *J. Cell Biol.* 194 61–75. 10.1083/jcb.201012074 21727194 PMC3135397

[B53] Goizet C. Boukhris A. Durr A. Beetz C. Truchetto J. Tesson C. (2009). CYP7B1 mutations in pure and complex forms of hereditary spastic paraplegia type 5. *Brain* 132 1589–1600. 10.1093/brain/awp073 19439420

[B54] Gomez-Ospina N. (2006). “Arylsulfatase a deficiency,” in *GeneReviews^®^*, eds Adam M. P. Ardinger H. H. Pagon R. A. Wallace S. E. Bean L. J. Stephens K. (Seattle, WA: University of Washington, Seattle). 20301309

[B55] Green J. T. Orr S. K. Bazinet R. P. (2008). The emerging role of group VI calcium-independent phospholipase A2 in releasing docosahexaenoic acid from brain phospholipids: Fig. 1. *J. Lipid Res.* 49 939–944. 10.1194/jlr.R700017-JLR200 18252846

[B56] Griffiths I. (1998). Axonal swellings and degeneration in mice lacking the major proteolipid of myelin. *Science* 280 1610–1613. 10.1126/science.280.5369.1610 9616125

[B57] Hama K. Fujiwara Y. Morita M. Yamazaki F. Nakashima Y. Takei S. (2018). Profiling and imaging of phospholipids in brains of *Abcd1*-deficient mice. *Lipids* 53 85–102. 10.1002/lipd.12022 29469952

[B58] Hammer M. B. Eleuch-Fayache G. Schottlaender L. V. Nehdi H. Gibbs J. R. Arepalli S. K. (2013). Mutations in GBA2 cause autosomal-recessive cerebellar ataxia with spasticity. *Am. J. Hum. Genet.* 92 245–251. 10.1016/j.ajhg.2012.12.012 23332917 PMC3567281

[B59] Hanein S. Martin E. Boukhris A. Byrne P. Goizet C. Hamri A. (2008). Identification of the SPG15 gene, encoding spastizin, as a frequent cause of complicated autosomal-recessive spastic paraplegia, including kjellin syndrome. *Am. J. Hum. Genet.* 82 992–1002. 10.1016/j.ajhg.2008.03.004 18394578 PMC2427184

[B60] Harding A. E. (1983). Classification of the hereditary ataxias and paraplegias. *Lancet Lond. Engl.* 1 1151–1155. 10.1016/s0140-6736(83)928799 6133167

[B61] Harlalka G. V. Lehman A. Chioza B. Baple E. L. Maroofian R. Cross H. (2013). Mutations in B4GALNT1 (GM2 synthase) underlie a new disorder of ganglioside biosynthesis. *Brain* 136 3618–3624. 10.1093/brain/awt270 24103911 PMC3859217

[B62] Hehr U. Bauer P. Winner B. Schule R. Olmez A. Koehler W. (2007). Long-term course and mutational spectrum of spatacsin-linked spastic paraplegia. *Ann. Neurol.* 62 656–665. 10.1002/ana.21310 18067136

[B63] Hein S. Schonfeld P. Kahlert S. Reiser G. (2008). Toxic effects of X-linked adrenoleukodystrophy-associated, very long chain fatty acids on glial cells and neurons from rat hippocampus in culture. *Hum. Mol. Genet.* 17 1750–1761. 10.1093/hmg/ddn066 18344355

[B64] Hess B. Saftig P. Hartmann D. Coenen R. Lullmann-Rauch R. Goebel H. H. (1996). Phenotype of arylsulfatase A-deficient mice: relationship to human metachromatic leukodystrophy. *Proc. Natl. Acad. Sci. U.S.A.* 93 14821–14826. 10.1073/pnas.93.25.14821 8962139 PMC26220

[B65] Higgs H. N. Han M. H. Johnson G. E. Glomset J. A. (1998). Cloning of a phosphatidic acid-preferring phospholipase A1 from bovine testis. *J. Biol. Chem.* 273 5468–5477. 10.1074/jbc.273.10.5468 9488669

[B66] Hirst J. Edgar J. R. Esteves T. Darios F. Madeo M. Chang J. (2015). Loss of AP-5 results in accumulation of aberrant endolysosomes: defining a new type of lysosomal storage disease. *Hum. Mol. Genet.* 24 4984–4996. 10.1093/hmg/ddv220 26085577 PMC4527494

[B67] Hirst J. Madeo M. Smets K. Edgar J. R. Schols L. Li J. (2016). Complicated spastic paraplegia in patients with *AP5Z1* mutations (SPG48). *Neurol. Genet.* 2:e98. 10.1212/NXG.0000000000000098 27606357 PMC5001803

[B68] Hooper C. Puttamadappa S. S. Loring Z. Shekhtman A. Bakowska J. C. (2010). Spartin activates atrophin-1-interacting protein 4 (AIP4) E3 ubiquitin ligase and promotes ubiquitination of adipophilin on lipid droplets. *BMC Biol.* 8:72. 10.1186/1741-7007-872 20504295 PMC2887783

[B69] Horibata Y. Elpeleg O. Eran A. Hirabayashi Y. Savitzki D. Tal G. (2018). EPT1 (selenoprotein I) is critical for the neural development and maintenance of plasmalogen in humans. *J. Lipid Res.* 59 1015–1026. 10.1194/jlr.P081620 29500230 PMC5983406

[B70] Huber M. D. Vesely P. W. Datta K. Gerace L. (2013). Erlins restrict SREBP activation in the ER and regulate cellular cholesterol homeostasis. *J. Cell Biol.* 203 427–436. 10.1083/jcb.201305076 24217618 PMC3824017

[B71] Huffnagel I. C. Dijkgraaf M. G. W. Janssens G. E. van Weeghel M. van Geel B. M. Poll-The B. T. (2019). Disease progression in women with X-linked adrenoleukodystrophy is slow. *Orphanet J. Rare Dis.* 14:30. 10.1186/s13023-019-10086 30732635 PMC6367840

[B72] Igisu H. Shimomura K. Kishimoto Y. Suzuki K. (1983). Lipids of developing brain of twitcher mouse. An authentic murine model of human Krabbe disease. *Brain J. Neurol.* 106(Pt 2), 405–417. 10.1093/brain/106.2.405 6850275

[B73] Igisu H. Suzuki K. (1984). Progressive accumulation of toxic metabolite in a genetic leukodystrophy. *Science* 224 753–755. 10.1126/science.6719111 6719111

[B74] Inamura N. Kito M. Go S. Kishi S. Hosokawa M. Asai K. (2018). Developmental defects and aberrant accumulation of endogenous psychosine in oligodendrocytes in a murine model of Krabbe disease. *Neurobiol. Dis.* 120 51–62. 10.1016/j.nbd.2018.08.023 30176352

[B75] Inloes J. M. Hsu K.-L. Dix M. M. Viader A. Masuda K. Takei T. (2014). The hereditary spastic paraplegia-related enzyme DDHD2 is a principal brain triglyceride lipase. *Proc. Natl. Acad. Sci. U.S.A.* 111 14924–14929. 10.1073/pnas.1413706111 25267624 PMC4205627

[B76] Inloes J. M. Jing H. Cravatt B. F. (2018). The spastic paraplegia-associated phospholipase DDHD1 is a primary brain phosphatidylinositol lipase. *Biochemistry* 57 5759–5767. 10.1021/acs.biochem.8b00810 30221923 PMC6237197

[B77] Inoue H. Baba T. Sato S. Ohtsuki R. Takemori A. Watanabe T. (2012). Roles of SAM and DDHD domains in mammalian intracellular phospholipase A1 KIAA0725p. *Biochim. Biophys. Acta BBA – Mol. Cell Res.* 1823 930–939. 10.1016/j.bbamcr.2012.02.002 22922100

[B78] Inoue K. Kubota S. Seyama Y. (1999). Cholestanol induces apoptosis of cerebellar neuronal cells. *Biochem. Biophys. Res. Commun.* 256 198–203. 10.1006/bbrc.1998.9497 10066446

[B79] Jakobsson A. Westerberg R. Jacobsson A. (2006). Fatty acid elongases in mammals: their regulation and roles in metabolism. *Prog. Lipid Res.* 45 237–249. 10.1016/j.plipres.2006.01.004 16564093

[B80] James P. F. Zoeller R. A. (1997). Isolation of animal cell mutants defective in long-chain fatty aldehyde dehydrogenase: sensitivity to fatty aldehydes and schiff’s base modification of phospholipids: implications for sjögren-larsson syndrome. *J. Biol. Chem.* 272 23532–23539. 10.1074/jbc.272.38.23532 9295289

[B81] Joshi D. C. Bakowska J. C. (2011). SPG20 protein spartin associates with cardiolipin via its plant-related senescence domain and regulates mitochondrial Ca2+ homeostasis. *PLoS ONE* 6:e19290. 10.1371/journal.pone.0019290 21559443 PMC3084803

[B82] Kameoka S. Adachi Y. Okamoto K. Iijima M. Sesaki H. (2018). Phosphatidic acid and cardiolipin coordinate mitochondrial dynamics. *Trends Cell Biol.* 28 67–76. 10.1016/j.tcb.2017.08.011 28911913 PMC5742555

[B83] Kanamori A. Nakayama J. Fukuda M. N. Stallcup W. B. Sasaki K. Fukuda M. (1997). Expression cloning and characterization of a cDNA encoding a novel membrane protein required for the formation of O-acetylated ganglioside: a putative acetyl-CoA transporter. *Proc. Natl. Acad. Sci. U.S.A.* 94 2897–2902. 10.1073/pnas.94.7.2897 9096318 PMC20294

[B84] Kanetake T. Sassa T. Nojiri K. Sawai M. Hattori S. Miyakawa T. (2019). Neural symptoms in a gene knockout mouse model of Sjögren-Larsson syndrome are associated with a decrease in 2-hydroxygalactosylceramide. *FASEB J.* 33 928–941. 10.1096/fj.201800291R 30085884

[B85] Kemp S. Berger J. Aubourg P. (2012). X-linked adrenoleukodystrophy: clinical, metabolic, genetic and pathophysiological aspects. *Biochim. Biophys. Acta BBA – Mol. Basis Dis.* 1822 1465–1474. 10.1016/j.bbadis.2012.03.012 22483867

[B86] Khundadze M. Kollmann K. Koch N. Biskup C. Nietzsche S. Zimmer G. (2013). A hereditary spastic paraplegia mouse model supports a role of ZFYVE26/SPASTIZIN for the endolysosomal system. *PLoS Genet.* 9:e1003988. 10.1371/journal.pgen.1003988 24367272 PMC3868532

[B87] Khundadze M. Ribaudo F. Hussain A. Rosentreter J. Nietzsche S. Thelen M. (2019). A mouse model for SPG48 reveals a block of autophagic flux upon disruption of adaptor protein complex five. *Neurobiol. Dis.* 127 419–431. 10.1016/j.nbd.2019.03.026 30930081

[B88] Klemm R. W. Norton J. P. Cole R. A. Li C. S. Park S. H. Crane M. M. (2013). A conserved role for atlastin GTPases in regulating lipid droplet size. *Cell Rep.* 3 1465–1475. 10.1016/j.celrep.2013.04.015 23684613 PMC3742324

[B89] Kobayashi T. Shinnoh N. Goto I. Kuroiwa Y. (1985). Hydrolysis of galactosylceramide is catalyzed by two genetically distinct acid beta-galactosidases. *J. Biol. Chem.* 260 14982–14987. 3934152

[B90] Kruer M. C. Paisán-Ruiz C. Boddaert N. Yoon M. Y. Hama H. Gregory A. (2010). Defective FA2H leads to a novel form of neurodegeneration with brain iron accumulation (NBIA). *Ann. Neurol.* 68 611–618. 10.1002/ana.22122 20853438 PMC6059612

[B91] Kruska N. Schönfeld P. Pujol A. Reiser G. (2015). Astrocytes and mitochondria from adrenoleukodystrophy protein (ABCD1)-deficient mice reveal that the adrenoleukodystrophy-associated very long-chain fatty acids target several cellular energy-dependent functions. *Biochim. Biophys. Acta BBA – Mol. Basis Dis.* 1852 925–936. 10.1016/j.bbadis.2015.01.005 25583114

[B92] Kutkowska-Kaźmierczak A. Rydzanicz M. Chlebowski A. Kłosowska-Kosicka K. Mika A. Gruchota J. (2018). Dominant ELOVL1 mutation causes neurological disorder with ichthyotic keratoderma, spasticity, hypomyelination and dysmorphic features. *J. Med. Genet.* 55 408–414. 10.1136/jmedgenet-2017-105172 29496980

[B93] Laule C. Vavasour I. M. Shahinfard E. Mädler B. Zhang J. Li D. K. B. (2018). Hematopoietic stem cell transplantation in late-onset krabbe disease: no evidence of worsening demyelination and axonal loss 4 years post-allograft. *J. Neuroimaging* 28 252–255. 10.1111/jon.12502 29479774

[B94] Laurenzi V. D. Rogers G. R. Hamrock D. J. Marekov L. N. Steinert P. M. Compton J. G. (1996). Sjögren–Larsson syndrome is caused by mutations in the fatty aldehyde dehydrogenase gene. *Nat. Genet.* 12 52–57. 10.1038/ng0196-52 8528251

[B95] Li W. Sandhoff R. Kono M. Zerfas P. Hoffmann V. Ding B. C.-H. (2007). Depletion of ceramides with very long chain fatty acids causes defective skin permeability barrier function, and neonatal lethality in ELOVL4 deficient mice. *Int. J. Biol. Sci.* 3 120–128. 10.7150/ijbs.3.120 17311087 PMC1796950

[B96] Lin P. Li J. Liu Q. Mao F. Li J. Qiu R. (2008). A missense mutation in slc33a1, which encodes the Acetyl-CoA transporter, causes autosomal-dominant spastic paraplegia (SPG42). *Am. J. Hum. Genet.* 83 752–759. 10.1016/j.ajhg.2008.11.003 19061983 PMC2668077

[B97] Liu P. Jiang B. Ma J. Lin P. Zhang Y. Shao C. (2017). S113R mutation in SLC33A1 leads to neurodegeneration and augmented BMP signaling in a mouse model. *Dis. Model. Mech.* 10 53–62. 10.1242/dmm.026880 27935820 PMC5278525

[B98] Lossos A. Elazar N. Lerer I. Schueler-Furman O. Fellig Y. Glick B. (2015). Myelin-associated glycoprotein gene mutation causes Pelizaeus-Merzbacher disease-like disorder. *Brain* 138 2521–2536. 10.1093/brain/awv204 26179919 PMC4643626

[B99] Lüders K. A. Patzig J. Simons M. Nave K.-A. Werner H. B. (2017). Genetic dissection of oligodendroglial and neuronal *Plp1* function in a novel mouse model of spastic paraplegia type 2. *Glia* 65 1762–1776. 10.1002/glia.23193 28836307

[B100] Ma J. Wang L. Yang Y.-M. Mao C.-H. Wan X.-H. (2018). Novel SERAC1 mutations in a Chinese patient presenting with parkinsonism and dystonia. *Neurol. Sci.* 39 967–969. 10.1007/s10072-018-3247-z 29332177

[B101] Marelli C. Lamari F. Rainteau D. Lafourcade A. Banneau G. Humbert L. (2018). Plasma oxysterols: biomarkers for diagnosis and treatment in spastic paraplegia type 5. *Brain* 141 72–84. 10.1093/brain/awx297 29228183

[B102] Martin E. Schüle R. Smets K. Rastetter A. Boukhris A. Loureiro J. L. (2013). Loss of function of glucocerebrosidase GBA2 is responsible for motor neuron defects in hereditary spastic paraplegia. *Am. J. Hum. Genet.* 92 238–244. 10.1016/j.ajhg.2012.11.021 23332916 PMC3567271

[B103] Maruyama T. Baba T. Maemoto Y. Hara-Miyauchi C. Hasegawa-Ogawa M. Okano H. J. (2018). Loss of DDHD2, whose mutation causes spastic paraplegia, promotes reactive oxygen species generation and apoptosis. *Cell Death Dis.* 9:797. 10.1038/s41419-018-0815-3 30038238 PMC6056544

[B104] Mehl E. Jatzkewitz H. (1968). Cerebroside 3-sulfate as a physiological substrate of arylsulfatase A. *Biochim. Biophys. Acta BBA – Enzymol.* 151 619–627. 10.1016/0005-2744(68)90008-9 5646041

[B105] Mignarri A. Magni A. Del Puppo M. Gallus G. N. Björkhem I. Federico A. (2016). Evaluation of cholesterol metabolism in cerebrotendinous xanthomatosis. *J. Inherit. Metab. Dis.* 39 75–83. 10.1007/s10545-015-98731 26153518

[B106] Mileykovskaya E. Dowhan W. (2014). Cardiolipin-dependent formation of mitochondrial respiratory supercomplexes. *Chem. Phys. Lipids* 179 42–48. 10.1016/j.chemphyslip.2013.10.012 24220496 PMC3947694

[B107] Mishra H. K. Prots I. Havlicek S. Kohl Z. Perez-Branguli F. Boerstler T. (2016). GSK3ß-dependent dysregulation of neurodevelopment in SPG11-patient induced pluripotent stem cell model: neurodevelopmental defects in motor neuron disease. *Ann. Neurol.* 79 826–840. 10.1002/ana.24633 26971897 PMC5084783

[B108] Mitomo H. Chen W.-H. Regen S. L. (2009). Oxysterol-induced rearrangement of the liquid-ordered phase: a possible link to Alzheimer’s disease? *J. Am. Chem. Soc.* 131 12354–12357. 10.1021/ja904308y 19658396 PMC2748238

[B109] Mochel F. (2018). Lipids and synaptic functions. *J. Inherit. Metab. Dis.* 41 1117–1122. 10.1007/s10545-018-02041 29869164

[B110] Montecchiani C. Pedace L. Lo Giudice T. Casella A. Mearini M. Gaudiello F. (2016). ALS5/SPG11/*KIAA1840* mutations cause autosomal recessive axonal Charcot–Marie–Tooth disease. *Brain* 139 73–85. 10.1093/brain/awv320 26556829 PMC5839554

[B111] Mosser J. Douar A.-M. Sarde C.-O. Kioschis P. Feil R. Moser H. (1993). Putative X-linked adrenoleukodystrophy gene shares unexpected homology with ABC transporters. *Nature* 361 726–730. 10.1038/361726a0 8441467

[B112] Mueller N. Sassa T. Morales-Gonzalez S. Schneider J. Salchow D. J. Seelow D. (2019). De novo mutation in *ELOVL1* causes ichthyosis, *acanthosis nigricans*, hypomyelination, spastic paraplegia, high frequency deafness and optic atrophy. *J. Med. Genet.* 56 164–175. 10.1136/jmedgenet-2018-105711 30487246

[B113] Murakami Y. Tawamie H. Maeda Y. Büttner C. Buchert R. Radwan F. (2014). Null mutation in PGAP1 impairing gpi-anchor maturation in patients with intellectual disability and encephalopathy. *PLoS Genet.* 10:e1004320. 10.1371/journal.pgen.1004320 24784135 PMC4006728

[B114] Nie S. Chen G. Cao X. Zhang Y. (2014). Cerebrotendinous xanthomatosis: a comprehensive review of pathogenesis, clinical manifestations, diagnosis, and management. *Orphanet J. Rare Dis.* 9:179. 10.1186/s13023-014-01794 25424010 PMC4264335

[B115] Novarino G. Fenstermaker A. G. Zaki M. S. Hofree M. Silhavy J. L. Heiberg A. D. (2014). Exome sequencing links corticospinal motor neuron disease to common neurodegenerative disorders. *Science* 343 506–511. 10.1126/science.1247363 24482476 PMC4157572

[B116] Ofman R. Dijkstra I. M. E. van Roermund C. W. T. Burger N. Turkenburg M. van Cruchten A. (2010). The role of ELOVL1 in very long-chain fatty acid homeostasis and X-linked adrenoleukodystrophy. *EMBO Mol. Med.* 2 90–97. 10.1002/emmm.201000061 20166112 PMC3377275

[B117] Olzmann J. A. Carvalho P. (2019). Dynamics and functions of lipid droplets. *Nat. Rev. Mol. Cell Biol.* 20 137–155. 10.1038/s41580-018-0085-z 30523332 PMC6746329

[B118] Ong W.-Y. Yeo J.-F. Ling S.-F. Farooqui A. A. (2005). Distribution of calcium-independent phospholipase A2 (iPLA2) in monkey brain. *J. Neurocytol.* 34 447–458. 10.1007/s11068-006-87304 16902765

[B119] Orlacchio A. Babalini C. Borreca A. Patrono C. Massa R. Basaran S. (2010). SPATACSIN mutations cause autosomal recessive juvenile amyotrophic lateral sclerosis. *Brain* 133 591–598. 10.1093/brain/awp325 20110243 PMC2822627

[B120] Orsini J. J. Escolar M. L. Wasserstein M. P. Caggana M. (2000). “Krabbe disease,” in *GeneReviews^®^*, eds Adam M. P. Ardinger H. H. Pagon R. A. Wallace S. E. Bean L. J. Stephens K. (Seattle, WA: University of Washington, Seattle). 20301416

[B121] Orthmann-Murphy J. L. Salsano E. Abrams C. K. Bizzi A. Uziel G. Freidin M. M. (2009). Hereditary spastic paraplegia is a novel phenotype for GJA12/GJC2 mutations. *Brain* 132 426–438. 10.1093/brain/awn328 19056803 PMC2640216

[B122] Ozaki K. Doi H. Mitsui J. Sato N. Iikuni Y. Majima T. (2015). A novel mutation in ELOVL4 leading to spinocerebellar ataxia (SCA) with the hot cross bun sign but lacking erythrokeratodermia: a broadened spectrum of SCA34. *JAMA Neurol.* 72 797–805. 10.1001/jamaneurol.2015.0610 26010696

[B123] Pan B. Fromholt S. E. Hess E. J. Crawford T. O. Griffin J. W. Sheikh K. A. (2005). Myelin-associated glycoprotein and complementary axonal ligands, gangliosides, mediate axon stability in the CNS and PNS: neuropathology and behavioral deficits in single- and double-null mice. *Exp. Neurol.* 195 208–217. 10.1016/j.expneurol.2005.04.017 15953602 PMC1852502

[B124] Papadopoulos C. Orso G. Mancuso G. Herholz M. Gumeni S. Tadepalle N. (2015). Spastin binds to lipid droplets and affects lipid metabolism. *PLoS Genet.* 11:e1005149. 10.1371/journal.pgen.1005149 25875445 PMC4395272

[B125] Park S. H. Zhu P.-P. Parker R. L. Blackstone C. (2010). Hereditary spastic paraplegia proteins REEP1, spastin, and atlastin-1 coordinate microtubule interactions with the tubular ER network. *J. Clin. Invest.* 120 1097–1110. 10.1172/JCI40979 20200447 PMC2846052

[B126] Parkinson-Lawrence E. J. Shandala T. Prodoehl M. Plew R. Borlace G. N. Brooks D. A. (2010). Lysosomal storage disease: revealing lysosomal function and physiology. *Physiology* 25 102–115. 10.1152/physiol.00041.2009 20430954

[B127] Pearce M. M. P. Wormer D. B. Wilkens S. Wojcikiewicz R. J. H. (2009). An endoplasmic reticulum (ER) membrane complex composed of SPFH1 and SPFH2 mediates the er-associated degradation of inositol 1,4,5-trisphosphate receptors. *J. Biol. Chem.* 284 10433–10445. 10.1074/jbc.M809801200 19240031 PMC2667730

[B128] Pennetta G. Welte M. A. (2018). Emerging links between lipid droplets and motor neuron diseases. *Dev. Cell* 45 427–432. 10.1016/j.devcel.2018.05.002 29787708 PMC5988256

[B129] Pérez-Brangulí F. Buchsbaum I. Y. Pozner T. Regensburger M. Fan W. Schray A. (2019). Human SPG11 cerebral organoids reveal cortical neurogenesis impairment. *Hum. Mol. Genet.* 28 961–971. 10.1093/hmg/ddy397 30476097 PMC6400051

[B130] Potter K. A. Kern M. J. Fullbright G. Bielawski J. Scherer S. S. Yum S. W. (2011). Central nervous system dysfunction in a mouse model of Fa2h deficiency. *Glia* 59 1009–1021. 10.1002/glia.21172 21491498 PMC3094470

[B131] Rafi M. A. Luzi P. Chen Y. Q. Wenger D. A. (1995). A large deletion together with a point mutation in the GALC gene is a common mutant allele in patients with infantile Krabbe disease. *Hum. Mol. Genet.* 4 1285–1289. 10.1093/hmg/4.8.1285 7581365

[B132] Rainier S. Bui M. Mark E. Thomas D. Tokarz D. Ming L. (2008). Neuropathy target esterase gene mutations cause motor neuron disease. *Am. J. Hum. Genet.* 82 780–785. 10.1016/j.ajhg.2007.12.018 18313024 PMC2427280

[B133] Raju D. Schonauer S. Hamzeh H. Flynn K. C. Bradke F. vom Dorp K. (2015). Accumulation of glucosylceramide in the absence of the beta-glucosidase GBA2 alters cytoskeletal dynamics. *PLoS Genet.* 11:e1005063. 10.1371/journal.pgen.1005063 25803043 PMC4372435

[B134] Ramirez A. Heimbach A. Gründemann J. Stiller B. Hampshire D. Cid L. P. (2006). Hereditary parkinsonism with dementia is caused by mutations in ATP13A2, encoding a lysosomal type 5 P-type ATPase. *Nat. Genet.* 38 1184–1191. 10.1038/ng1884 16964263

[B135] Rattay T. W. Lindig T. Baets J. Smets K. Deconinck T. Söhn A. S. (2019). FAHN/SPG35: a narrow phenotypic spectrum across disease classifications. *Brain* 142 1561–1572. 10.1093/brain/awz102 31135052 PMC6536916

[B136] Raymond G. V. Aubourg P. Paker A. Escolar M. Fischer A. Blanche S. (2019). Survival and functional outcomes in boys with cerebral adrenoleukodystrophy with and without hematopoietic stem cell transplantation. *Biol. Blood Marrow Transplant.* 25 538–548. 10.1016/j.bbmt.2018.09.036 30292747

[B137] Read D. J. Li Y. Chao M. V. Cavanagh J. B. Glynn P. (2009). Neuropathy target esterase is required for adult vertebrate axon maintenance. *J. Neurosci.* 29 11594–11600. 10.1523/JNEUROSCI.3007-09.2009 19759306 PMC3849655

[B138] Renvoisé B. Chang J. Singh R. Yonekawa S. FitzGibbon E. J. Mankodi A. (2014). Lysosomal abnormalities in hereditary spastic paraplegia types SPG15 and SPG11. *Ann. Clin. Transl. Neurol.* 1 379–389. 10.1002/acn3.64 24999486 PMC4078876

[B139] Renvoisé B. Malone B. Falgairolle M. Munasinghe J. Stadler J. Sibilla C. (2016). *Reep1* null mice reveal a converging role for hereditary spastic paraplegia proteins in lipid droplet regulation. *Hum. Mol. Genet.* 25 5111–5125. 10.1093/hmg/ddw315 27638887 PMC6078631

[B140] Rinaldi C. Schmidt T. Situ A. J. Johnson J. O. Lee P. R. Chen K. (2015). Mutation in *CPT1C* Associated With Pure Autosomal Dominant Spastic Paraplegia. *JAMA Neurol.* 72:561. 10.1001/jamaneurol.2014.4769 25751282 PMC5612424

[B141] Rizzo W. B. Craft D. A. (1991). Sjögren-Larsson syndrome. Deficient activity of the fatty aldehyde dehydrogenase component of fatty alcohol:NAD+ oxidoreductase in cultured fibroblasts. *J. Clin. Invest.* 88 1643–1648. 10.1172/JCI115478 1939650 PMC295691

[B142] Roeben B. Schüle R. Ruf S. Bender B. Alhaddad B. Benkert T. (2018). SERAC1 deficiency causes complicated HSP: evidence from a novel splice mutation in a large family. *J. Med. Genet.* 55 39–47. 10.1136/jmedgenet-2017-104622 28916646

[B143] Rydning S. L. Dudesek A. Rimmele F. Funke C. Krüger S. Biskup S. (2018). A novel heterozygous variant in *ERLIN2* causes autosomal dominant pure hereditary spastic paraplegia. *Eur. J. Neurol.* 25 943–e71. 10.1111/ene.13625 29528531

[B144] Salen G. Shefer S. Berginer V. (1991). Biochemical abnormalities in cerebrotendinous xanthomatosis. *Dev. Neurosci.* 13 363–370. 10.1159/000112186 1817043

[B145] Salen G. Steiner R. D. (2017). Epidemiology, diagnosis, and treatment of cerebrotendinous xanthomatosis (CTX). *J. Inherit. Metab. Dis.* 40 771–781. 10.1007/s10545-017-00938 28980151

[B146] Salo V. T. Belevich I. Li S. Karhinen L. Vihinen H. Vigouroux C. (2016). Seipin regulates ER–lipid droplet contacts and cargo delivery. *EMBO J.* 35 2699–2716. 10.15252/embj.201695170 27879284 PMC5167346

[B147] Sassa T. Ohno Y. Suzuki S. Nomura T. Nishioka C. Kashiwagi T. (2013). Impaired epidermal permeability barrier in mice lacking Elovl1, the gene responsible for very-long-chain fatty acid production. *Mol. Cell. Biol.* 33 2787–2796. 10.1128/mcb.00192-13 23689133 PMC3700134

[B148] Saugier-Veber P. Munnich A. Bonneau D. Rozet J.-M. Le Merrer M. Gil R. (1994). X–linked spastic paraplegia and Pelizaeus–Merzbacher disease are allelic disorders at the proteolipid protein locus. *Nat. Genet.* 6 257–262. 10.1038/ng0394-257 8012387

[B149] Schicks J. Synofzik M. Pétursson H. Huttenlocher J. Reimold M. Schöls L. (2011). Atypical juvenile parkinsonism in a consanguineous *SPG15* family: letters to the editor. *Mov. Disord.* 26 565–566. 10.1002/mds.23472 21462267

[B150] Schiffmann R. FitzGibbon E. J. Harris C. DeVile C. Davies E. H. Abel L. (2008). Randomized, controlled trial of miglustat in Gaucher’s disease type 3. *Ann. Neurol.* 64 514–522. 10.1002/ana.21491 19067373 PMC2605167

[B151] Schnaar R. L. Lopez P. H. H. (2009). Myelin-associated glycoprotein and its axonal receptors. *J. Neurosci. Res.* 87 3267–3276. 10.1002/jnr.21992 19156870 PMC2892843

[B152] Schöls L. Rattay T. W. Martus P. Meisner C. Baets J. Fischer I. (2017). Hereditary spastic paraplegia type 5: natural history, biomarkers and a randomized controlled trial. *Brain* 140 3112–3127. 10.1093/brain/awx273 29126212 PMC5841036

[B153] Schüle R. Siddique T. Deng H.-X. Yang Y. Donkervoort S. Hansson M. (2010). Marked accumulation of 27-hydroxycholesterol in SPG5 patients with hereditary spastic paresis. *J. Lipid Res.* 51 819–823. 10.1194/jlr.M002543 19812052 PMC2842155

[B154] Schuurs-Hoeijmakers J. H. M. Geraghty M. T. Kamsteeg E.-J. Ben-Salem S. de Bot S. T. Nijhof B. (2012). Mutations in DDHD2, encoding an intracellular phospholipase A1, cause a recessive form of complex hereditary spastic paraplegia. *Am. J. Hum. Genet.* 91 1073–1081. 10.1016/j.ajhg.2012.10.017 23176823 PMC3516595

[B155] Sheikh K. A. Sun J. Liu Y. Kawai H. Crawford T. O. Proia R. L. (1999). Mice lacking complex gangliosides develop Wallerian degeneration and myelination defects. *Proc. Natl. Acad. Sci. U.S.A.* 96 7532–7537. 10.1073/pnas.96.13.7532 10377449 PMC22120

[B156] Siller M. Goyal S. Yoshimoto F. K. Xiao Y. Wei S. Guengerich F. P. (2014). Oxidation of endogenous *N*-arachidonoylserotonin by human cytochrome P450 2U1. *J. Biol. Chem.* 289 10476–10487. 10.1074/jbc.M114.550004 24563460 PMC4036169

[B157] Simons J. P. Al-Shawi R. Minogue S. Waugh M. G. Wiedemann C. Evangelou S. (2009). Loss of phosphatidylinositol 4-kinase 2α activity causes late onset degeneration of spinal cord axons. *Proc. Natl. Acad. Sci. U.S.A.* 106 11535–11539. 10.1073/pnas.0903011106 19581584 PMC2710652

[B158] Sjogren T. Larsson T. (1957). Oligophrenia in combination with congenital ichthyosis and spastic disorders; a clinical and genetic study. *Acta Psychiatr. Neurol. Scand. Suppl.* 113 1–112. 13457946

[B159] Soderblom C. Blackstone C. (2006). Traffic accidents: molecular genetic insights into the pathogenesis of the hereditary spastic paraplegias. *Pharmacol. Ther.* 109 42–56. 10.1016/j.pharmthera.2005.06.001 16005518

[B160] Stelten B. M. L. Huidekoper H. H. van de Warrenburg B. P. C. Brilstra E. H. Hollak C. E. M. Haak H. R. (2019). Long-term treatment effect in cerebrotendinous xanthomatosis depends on age at treatment start. *Neurology* 92 e83–e95. 10.1212/WNL.0000000000006731 30530799

[B161] Stevanin G. Azzedine H. Denora P. Boukhris A. Tazir M. Lossos A. (2008). Mutations in SPG11 are frequent in autosomal recessive spastic paraplegia with thin corpus callosum, cognitive decline and lower motor neuron degeneration. *Brain* 131 772–784. 10.1093/brain/awm293 18079167

[B162] Stevanin G. Rastetter A. Esteves T. Hanein S. Depienne C. Brice A. (2019). Dominant negative heterozygous mutation in Erlin2 prevents degradation of IP3 receptors and is responsible for hereditary spastic paraplegia 37. *Eur. J. Hum. Genet.* 1174–1813. 10.1038/s41431-019-0494231597946

[B163] Stevanin G. Santorelli F. M. Azzedine H. Coutinho P. Chomilier J. Denora P. S. (2007). Mutations in SPG11, encoding spatacsin, are a major cause of spastic paraplegia with thin corpus callosum. *Nat. Genet.* 39 366–372. 10.1038/ng1980 17322883

[B164] Sultana S. Reichbauer J. Schüle R. Mochel F. Synofzik M. van der Spoel A. C. (2015). Lack of enzyme activity in GBA2 mutants associated with hereditary spastic paraplegia/cerebellar ataxia (SPG46). *Biochem. Biophys. Res. Commun.* 465 35–40. 10.1016/j.bbrc.2015.07.112 26220345

[B165] Suzuki S. O. Iwaki T. Arakawa K. Furuya H. Fujii N. Iwaki A. (2011). An autopsy case of adult-onset hereditary spastic paraplegia type 2 with a novel mutation in exon 7 of the proteolipid protein 1 gene. *Acta Neuropathol. (Berl.)* 122 775–781. 10.1007/s00401-011-0916-x 22101368

[B166] Svennerholm L. Vanier M. T. Månsson J. E. (1980). Krabbe disease: a galactosylsphingosine (psychosine) lipidosis. *J. Lipid Res.* 21 53–64. 7354254

[B167] Synofzik M. Hufnagel R. B. Züchner S. (2014). “PNPLA6-related disorders,” in *GeneReviews^®^*, eds Adam M. P. Ardinger H. H. Pagon R. A. Wallace S. E. Bean L. J. Stephens K. (Seattle, WA: University of Washington, Seattle).

[B168] Szymanski K. M. Binns D. Bartz R. Grishin N. V. Li W.-P. Agarwal A. K. (2007). The lipodystrophy protein seipin is found at endoplasmic reticulum lipid droplet junctions and is important for droplet morphology. *Proc. Natl. Acad. Sci. U.S.A.* 104 20890–20895. 10.1073/pnas.0704154104 18093937 PMC2409237

[B169] Takahashi H. Suzuki K. (1984). Demyelination in the spinal cord of murine globoid cell leukodystrophy (the twitcher mouse). *Acta Neuropathol. (Berl.)* 62 298–308. 10.1007/BF00687612 6730907

[B170] Tanaka S. Maeda Y. Tashima Y. Kinoshita T. (2004). Inositol deacylation of glycosylphosphatidylinositol-anchored proteins is mediated by mammalian PGAP1 and yeast Bst1p. *J. Biol. Chem.* 279 14256–14263. 10.1074/jbc.M313755200 14734546

[B171] Tesson C. Nawara M. Salih M. A. M. Rossignol R. Zaki M. S. Al Balwi M. (2012). Alteration of fatty-acid-metabolizing enzymes affects mitochondrial form and function in hereditary spastic paraplegia. *Am. J. Hum. Genet.* 91 1051–1064. 10.1016/j.ajhg.2012.11.001 23176821 PMC3516610

[B172] Theda C. Moser A. B. Powers J. M. Moser H. W. (1992). Phospholipids in X-linked adrenoleukodystrophy white matter: fatty acid abnormalities before the onset of demyelination. *J. Neurol. Sci.* 110 195–204. 10.1016/0022-510X(92)90028-J 1506859

[B173] Theofilopoulos S. Griffiths W. J. Crick P. J. Yang S. Meljon A. Ogundare M. (2014). Cholestenoic acids regulate motor neuron survival via liver X receptors. *J. Clin. Invest.* 124 4829–4842. 10.1172/JCI68506 25271621 PMC4347238

[B174] Trotta N. Orso G. Rossetto M. G. Daga A. Broadie K. (2004). The Hereditary Spastic Paraplegia Gene, Spastin, Regulates Microtubule Stability To Modulate Synaptic Structure And Function. *Curr. Biol.* 14 1135–1147. 10.1016/j.cub.2004.06.058 15242610

[B175] Tsaousidou M. K. Ouahchi K. Warner T. T. Yang Y. Simpson M. A. Laing N. G. (2008). Sequence alterations within CYP7B1 implicate defective cholesterol homeostasis in motor-neuron degeneration. *Am. J. Hum. Genet.* 82 510–515. 10.1016/j.ajhg.2007.10.001 18252231 PMC2426914

[B176] Twig G. Elorza A. Molina A. J. A. Mohamed H. Wikstrom J. D. Walzer G. (2008). Fission and selective fusion govern mitochondrial segregation and elimination by autophagy. *EMBO J.* 27 433–446. 10.1038/sj.emboj.7601963 18200046 PMC2234339

[B177] van Rappard D. F. Boelens J. J. van Egmond M. E. Kuball J. van Hasselt P. M. Oostrom K. J. (2016). Efficacy of hematopoietic cell transplantation in metachromatic leukodystrophy: the Dutch experience. *Blood* 127 3098–3101. 10.1182/blood-2016-03-708479 27118454

[B178] van Roermund C. W. T. Visser W. F. IJlst L. van Cruchten A. Boek M. Kulik W. (2008). The human peroxisomal ABC half transporter ALDP functions as a homodimer and accepts acyl-CoA esters. *FASEB J.* 22 4201–4208. 10.1096/fj.08-110866 18757502

[B179] Vantaggiato C. Panzeri E. Castelli M. Citterio A. Arnoldi A. Santorelli F. M. (2019). ZFYVE26/SPASTIZIN and SPG11/SPATACSIN mutations in hereditary spastic paraplegia types AR-SPG15 and AR-SPG11 have different effects on autophagy and endocytosis. *Autophagy* 15 34–57. 10.1080/15548627.2018.1507438 30081747 PMC6287682

[B180] Varga R.-E. Khundadze M. Damme M. Nietzsche S. Hoffmann B. Stauber T. (2015). In vivo evidence for lysosome depletion and impaired autophagic clearance in hereditary spastic paraplegia type SPG11. *PLoS Genet.* 11:e1005454. 10.1371/journal.pgen.1005454 26284655 PMC4540459

[B181] Waldhüter N. Köhler W. Hemmati P. G. Jehn C. Peceny R. Vuong G. L. (2019). Allogeneic hematopoietic stem cell transplantation with myeloablative conditioning for adult cerebral X-linked adrenoleukodystrophy. *J. Inherit. Metab. Dis.* 42 313–324. 10.1002/jimd.12044 30746707

[B182] Wang H. Lo W.-T. Haucke V. (2019). Phosphoinositide switches in endocytosis and in the endolysosomal system. *Curr. Opin. Cell Biol.* 59 50–57. 10.1016/j.ceb.2019.03.011 31029845

[B183] Watkins P. A. Gould S. J. Smith M. A. Braiterman L. T. Wei H. M. Kok F. (1995). Altered expression of ALDP in X-linked adrenoleukodystrophy. *Am. J. Hum. Genet.* 57 292–301. 7668254 PMC1801558

[B184] Wei S. Soh S. L.-Y. Xia J. Ong W.-Y. Pang Z. P. Han W. (2014). Motor neuropathy-associated mutation impairs Seipin functions in neurotransmission. *J. Neurochem.* 129 328–338. 10.1111/jnc.12638 24345054

[B185] Wittke D. Hartmann D. Gieselmann V. Lüllmann-Rauch R. (2004). Lysosomal sulfatide storage in the brain of arylsulfatase A-deficient mice: cellular alterations and topographic distribution. *Acta Neuropathol. (Berl.)* 108 261–271. 10.1007/s00401-004-08836 15322834

[B186] Wnętrzak A. Makyła-Juzak K. Filiczkowska A. Kulig W. Dynarowicz-Łątka P. (2017). Oxysterols versus cholesterol in model neuronal membrane. I. The case of 7-ketocholesterol, the langmuir monolayer study. *J. Membr. Biol.* 250 553–564. 10.1007/s00232-017-99848 28861595 PMC5613072

[B187] Woeste M. A. Stern S. Raju D. N. Grahn E. Dittmann D. Gutbrod K. (2019). Species-specific differences in nonlysosomal glucosylceramidase GBA2 function underlie locomotor dysfunction arising from loss-of-function mutations. *J. Biol. Chem.* 294 3853–3871. 10.1074/jbc.RA118.006311 30662006 PMC6422076

[B188] Wong J. C. Walsh K. Hayden D. Eichler F. S. (2018). Natural history of neurological abnormalities in cerebrotendinous xanthomatosis. *J. Inherit. Metab. Dis.* 41 647–656. 10.1007/s10545-018-01529 29484516

[B189] Wood P. L. Smith T. Pelzer L. Goodenowe D. B. (2011). Targeted metabolomic analyses of cellular models of pelizaeus-merzbacher disease reveal plasmalogen and myo-inositol solute carrier dysfunction. *Lipids Health Dis.* 10:102. 10.1186/1476-511X-10102 21682894 PMC3141545

[B190] Wortmann S. B. Vaz F. M. Gardeitchik T. Vissers L. E. L. M. Renkema G. H. Schuurs-Hoeijmakers J. H. M. (2012). Mutations in the phospholipid remodeling gene SERAC1 impair mitochondrial function and intracellular cholesterol trafficking and cause dystonia and deafness. *Nat. Genet.* 44 797–802. 10.1038/ng.2325 22683713

[B191] Yahalom G. Tsabari R. Molshatzki N. Ephraty L. Cohen H. Hassin-Baer S. (2013). Neurological outcome in cerebrotendinous xanthomatosis treated with chenodeoxycholic acid: early versus late diagnosis. *Clin. Neuropharmacol.* 36 78–83. 10.1097/WNF.0b013e318288076a 23673909

[B192] Yang L. J. Zeller C. B. Shaper N. L. Kiso M. Hasegawa A. Shapiro R. E. (1996). Gangliosides are neuronal ligands for myelin-associated glycoprotein. *Proc. Natl. Acad. Sci. U.S.A.* 93 814–818. 10.1073/pnas.93.2.814 8570640 PMC40139

[B193] Yao D. McGonigal R. Barrie J. A. Cappell J. Cunningham M. E. Meehan G. R. (2014). Neuronal expression of GalNAc transferase is sufficient to prevent the age-related neurodegenerative phenotype of complex ganglioside-deficient mice. *J. Neurosci.* 34 880–891. 10.1523/JNEUROSCI.3996-13.2014 24431446 PMC3891965

[B194] Yoshii Y. Furukawa T. Saga T. Fujibayashi Y. (2015). Acetate/acetyl-CoA metabolism associated with cancer fatty acid synthesis: overview and application. *Cancer Lett.* 356 211–216. 10.1016/j.canlet.2014.02.019 24569091

[B195] Yung Y. C. Stoddard N. C. Mirendil H. Chun J. (2015). Lysophosphatidic acid signaling in the nervous system. *Neuron* 85 669–682. 10.1016/j.neuron.2015.01.009 25695267 PMC4400838

[B196] Zhang K. Kniazeva M. Han M. Li W. Yu Z. Yang Z. (2001). A 5-bp deletion in ELOVL4 is associated with two related forms of autosomal dominant macular dystrophy. *Nat. Genet.* 27 89–93. 10.1038/83817 11138005

[B197] Zoller I. Meixner M. Hartmann D. Bussow H. Meyer R. Gieselmann V. (2008). Absence of 2-hydroxylated sphingolipids is compatible with normal neural development but causes late-onset axon and myelin sheath degeneration. *J. Neurosci.* 28 9741–9754. 10.1523/JNEUROSCI.0458-08.2008 18815260 PMC6671223

